# Functional Brain Connectivity during Multiple Motor Imagery Tasks in Spinal Cord Injury

**DOI:** 10.1155/2018/9354207

**Published:** 2018-05-02

**Authors:** Alkinoos Athanasiou, Nikos Terzopoulos, Niki Pandria, Ioannis Xygonakis, Nicolas Foroglou, Konstantinos Polyzoidis, Panagiotis D. Bamidis

**Affiliations:** ^1^Biomedical Electronics Robotics & Devices (BERD) Group, Lab of Medical Physics, School of Medicine, Faculty of Health Sciences, Aristotle University of Thessaloniki (AUTH), 54124 Thessaloniki, Greece; ^2^1st Department of Neurosurgery, “AHEPA” University General Hospital, Aristotle University of Thessaloniki (AUTH), 54636 Thessaloniki, Greece

## Abstract

Reciprocal communication of the central and peripheral nervous systems is compromised during spinal cord injury due to neurotrauma of ascending and descending pathways. Changes in brain organization after spinal cord injury have been associated with differences in prognosis. Changes in functional connectivity may also serve as injury biomarkers. Most studies on functional connectivity have focused on chronic complete injury or resting-state condition. In our study, ten right-handed patients with incomplete spinal cord injury and ten age- and gender-matched healthy controls performed multiple visual motor imagery tasks of upper extremities and walking under high-resolution electroencephalography recording. Directed transfer function was used to study connectivity at the cortical source space between sensorimotor nodes. Chronic disruption of reciprocal communication in incomplete injury could result in permanent significant decrease of connectivity in a subset of the sensorimotor network, regardless of positive or negative neurological outcome. Cingulate motor areas consistently contributed the larger outflow (right) and received the higher inflow (left) among all nodes, across all motor imagery categories, in both groups. Injured subjects had higher outflow from left cingulate than healthy subjects and higher inflow in right cingulate than healthy subjects. Alpha networks were less dense, showing less integration and more segregation than beta networks. Spinal cord injury patients showed signs of increased local processing as adaptive mechanism. This trial is registered with NCT02443558.

## 1. Introduction

Reciprocal communication of the central and peripheral nervous systems is compromised during spinal cord injury (SCI), a condition that often causes permanent disability due to massive neurotrauma of ascending and descending pathways [1, 2]. While changes in brain activity and brain organization may seem trivial, when compared to the underlying injury of the pathways, they have nevertheless been consistently associated with SCI [3–5]. Such changes have also been observed at the early stages after the injury and have been associated with differences regarding the prognosis of SCI patients' recovery [4–6]. Demonstrated structural changes of the brain include atrophy of afferent neural pathways, microstructural changes of efferent axons, and disorder of white matter integrity in multiple nodes of the sensorimotor cortex that involve the primary motor and somatosensory areas [7] and also diffuse neuronal degeneration [8].

Functional connectivity (FC) after SCI has been studied by means of electroencephalography (EEG) [9–15] and functional magnetic resonance imaging (fMRI) [6, 16–21]. Poor recovery after SCI has been associated with decreased FC strengths between midline sensorimotor network nodes during resting state, while the opposite pattern has been associated with good recovery [6]. Supplementary and cingulate motor areas have been shown to play important roles during the sensorimotor neurophysiological process [9, 11], while unique interactions and temporal dynamics have been identified in the functional networks of SCI patients [12, 14]. Connectivity changes have been hypothesized to be able to serve as injury biomarkers [22] while novel methods have been developed and have been proposed in order to study the brain's connectome following SCI in hopes of providing more reliable evidence of these changes [23].

Most studies on functional connectivity after SCI have focused on patients with chronic complete injury, including the majority of EEG studies. Only a couple of studies employing fMRI as a modality to detect brain activity have assessed patients with incomplete injury but those have only studied connectivity during resting-state condition so far [6, 16–18]. Moreover, the pioneer EEG studies on functional cortical connectivity of SCI patients during motor imagery have employed a robust but rather limited study design [9]. In this design, the motor imagery task involved an attempt to move the paralyzed right foot and was performed simultaneously with one motor execution task (lip protrusion), while the functional networks were subsequently analyzed using a variety of tools. So far, to the authors' best knowledge, no study has been performed employing multiple motor imagery tasks, especially of the upper extremities, aiming to analyze differences in the formed networks. Moreover, incomplete injury at the chronic phases remains understudied compared to chronic complete injury with regard to functional connectivity networks. Despite the clinical and social impact of SCI, so far published studies have been unable to form a complete model regarding the effect of the injury on brain networks, although effort has been made into modeling-specific aspects, like resting-state connectivity and chronic injury [3, 22]. It can be hypothesized—and there are also indications—that even incomplete spinal cord injury may show measurable effects on the functional sensorimotor network [24] that could be important for modeling the condition in relation to prognosis [3, 6, 16].

Motor imagery, apart from its importance to the study of brain activity after neurotrauma, has shown great potential in motor skill learning and in rehabilitation of upper and lower limb paralysis [25, 26]. It has been established that motor imagery produces patterns of brain activation and brain connectivity similar to those of motor execution [27, 28], while the visual motor imagery class also activates a distinct task-dependent neural system [29, 30]. Motor imagery has been used as a modality to induce plasticity and recovery in a range of conditions [31], including complete cervical spinal cord injury [32] and stroke [33]. Moreover, motor imagery has been also used as a control modality for brain-computer interface implementations of exoskeletons for complete [34] and incomplete spinal cord injury [35]. Functional recovery has been induced even in the case of complete injuries using such an approach [34]. Such results demonstrate the importance of motor imagery functional networks studies to accurately model the plastic changes that occur after SCI.

In our previous work, we have presented our study with a cohort of SCI patients and healthy control subjects that exercised motor imagery to achieve control of anthropomorphic robotic arms in various movement tasks. We have accounted for development [36], pilot experiments, and brain network analysis [37, 38], and we have presented a detailed user perception and performance assessment study, based on neurological and psychometric evaluation [39]. In the current paper, we present an elaborate analysis of the functional connectivity networks formed on the sensorimotor cortex during visual motor imagery of multiple motor tasks performed by subjects with SCI and healthy controls. In Materials and Methods, we briefly present the experimental setup and detail our signal processing computational workflow, network analysis, and statistical comparisons. In Results, we detail important findings with regard to the effect of injury, motor imagery category, brainwave rhythm, and timing of imagery. In Discussion, we attempt to interpret our results in the context of already published studies in the field, and we note the limitations of this approach.

## 2. Materials and Methods

### 2.1. Experimental Setup

#### 2.1.1. Recruitment and Subject Assessment

The experimental setup has been previously described in detail, including subject assessment [39] and procedures [36, 37], so we will hereby provide only a brief overview. Our experimental protocol was approved by the institutional ethical committee [40], and all subjects signed an informed consent form. We recruited 8 male and 2 female patients with SCI (age: mean 46.0, SD 17.64, range 28–74 years) and ten gender- and age-matched healthy controls. All participants were right-handed and reported no prior experience in mental imagery (Table 1).

For both groups, we collected demographics and medical history; also, a specialist physician performed neurological examination using the International Standards for Neurological Classification of Spinal Cord Injury, to document classification in American Spinal Injury Association Impairment Scale (AIS) and the Neurological Level of Injury (NLI). Subject assessment also included subjective reporting of imagery capacity, using Vividness of Visual Imagery Questionnaire (VVIQ) [41] with eyes open (Table 1). Within the SCI group, 60% of the patients were grouped into positive outcome based on the neurological assessment (4 AIS D, 2 AIS E), and 40% of the patients were grouped into negative outcome (1 AIS A, 2 AIS B, and 1 AIS C).

#### 2.1.2. Experimental Procedure

The experiment took place inside a magnetic shielded room for EEG recording, specially designed for presentation capability and audiovisual monitoring of the participants. The subjects sat at a 1 m distance across a 21^″^ computer monitor. They wore an active electrode cap (Brain Products, Germany) and were connected to a 128-channel EEG (Nihon-Kohden, Japan) according to the high-resolution EEG 10–5 international electrode system [42]. Recordings were taken at a sampling rate of 1000 Hz and an impedance threshold of 10 kohm [36]. Initially, we recorded resting-state activity, 1.5 min with open eyes and 1.5 min with closed eyes.

**Figure 1:**
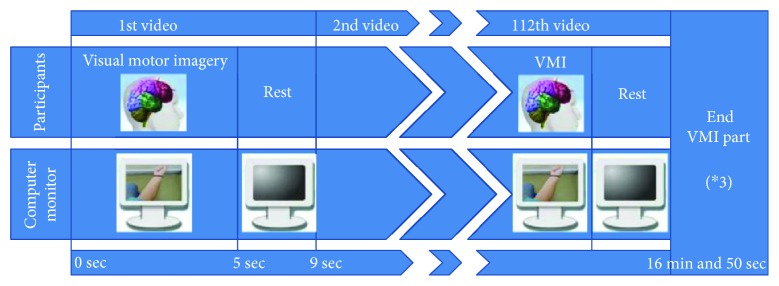
Flow diagram of the experimental procedure of one part of the visual motor imagery presentation. Each presented video lasted 5 seconds and was followed by 4 seconds of black resting screen. The videos were presented 9 times each, in a random order. The presentation was divided into three parts, lasting approximately 17 minutes each, with an intermission between them.

In the main experimental part, the subjects watched videos of 32 different arm motor tasks, a walking task video, and an oddball video. The videos were presented in random order. All arm motor task videos were presented from the perspective of the participant watching his or her own arms and were gender-matched. The walking task video presented a pair of gender-matched legs walking, while seeing them from the perspective of watching one's own legs. The oddball video showed a wildlife documentary. The videos lasted 5 sec, each followed by a black screen with duration of 4 sec. A trigger channel was recorded at the onset of each visual cue (the start of each video) through an optic diode. The presentation was separated in three parts of about 17 min each, with 5 min of rest between them (Figure 1). At conclusion, each task had been presented 9 times in total. The subjects were asked to perform visual motor imagery (VMI), while watching a motor task, without actually moving their limbs (regardless of neurological status or group) and were instructed to rest while watching the black screen. They already knew that the videos would be presented at random but not of the presence of an oddball video (video showing wildlife). Moreover, the subjects' arms, torso, and legs were covered with a black curtain during the whole procedure in order to facilitate mental registration of the presented arms and legs into their perceived body schema [43].

The 32 upper extremity motor tasks consisted of 8 independent movements (degrees of freedom) ^∗^ 2 directions of movement ^∗^ 2 extremities, comprising the full range of motion of the human arms and were classified into categories for further analysis [39]. In short, the 8 categories of motor tasks were “Hands,” “Left,” “Right,” “Proximal,” “Distal,” “Rotational,” “Linear,” and “Walking.”

The “Hands” category included all 32 tasks of both upper extremities. The “Left” and “Right” categories included 16 motor tasks each of the respective upper extremity (left or right). The “Proximal” category included 16 motor tasks of the shoulder and elbow joints of both extremities, while the “Distal” category included the remaining 16 motor tasks of wrist joints and fingers. Further, the “Rotational” category included those 8 motor tasks that result in rotational motion, and the “Linear” category included those 24 motor tasks of both extremities that resulted in linear motion (Table 2). Finally, the “Walking” category was also defined as a separate category of motor imagery, consisting only of the walking motor imagery task, for a total of 8 categories. Table 2 lists in summary all presented motor tasks of one upper extremity (16 tasks), for each showing the degree of freedom, direction of movement, classification by proximity, and resulted motion.

### 2.2. Signal Analysis

#### 2.2.1. Signal Preprocessing

**Figure 2:**
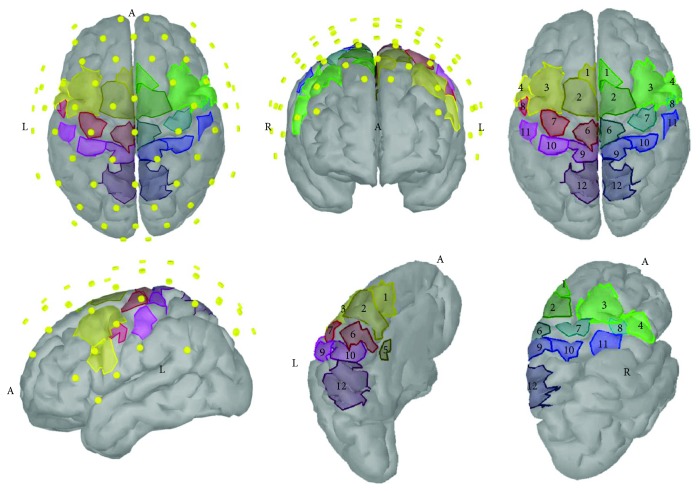
Regions of interest (ROIs) of the sensorimotor cortex and the overlying subset of electrodes that was used for signal analysis in our study. 1: presupplementary motor area (pSMA); 2: supplementary motor area (SMA); 3: dorsal premotor area (PMd); 4: ventral premotor area (PMv); 5: cingulate motor area (CMA); 6: primary foot motor area (M1F); 7: primary hand motor area (M1H); 8: primary lip motor area (M1L); 9: primary foot somatosensory area (S1F); 10: primary hand somatosensory area (S1H); 11: secondary somatosensory area (S2); 12: somatosensory association area (SAC).

Signal analysis was performed on a subset of the 10–5 international electrode system that is overlying the cortical sensorimotor areas [44] that were later defined as regions of interest (ROIs) for this study (Figure 2). This subset included 64 electrodes: AFF5h, AFF3h, AFF1h, AFz, AFF2h, AFF4h, AFF6h, F5, F3, F1, Fz, F2, F4, F6, FFT7h, FFC3h, FFC1h, FFC2h, FFC4h, FFT8h, FT7, FC5, FC3, FC1, FC2, FC4, FC6, FT8, FTT7h, FCC5h, FCC3h, FCC1h, FCC4h, FCC6h, FTT8h, C5, C3, C1, Cz, C2, C4, C6, CCP3h, CCP1h, CCP2h, CCP4h, CP5, CP3, CP1, CPz, CP2, CP4, CP6, CPP3h, CPP1h, CPP2h, CPP4h, P3, P1, Pz, P2, P4, PPO1h, and PPO2h. As scalp electrodes capture mixed activity from unknown cortical and subcortical brain sources, recording brain activity related to motor tasks only from the sensors overlying the sensorimotor area presents some risk for loss of information but also presents certain advantages. This approach has been used in EEG source imaging studies regarding motor tasks with good results [45–48], as the signal of interest is less attenuated and signal to noise ratio is higher than in distant sensors, while contaminated channels closer to muscular artifact generators are excluded.

**Figure 3:**
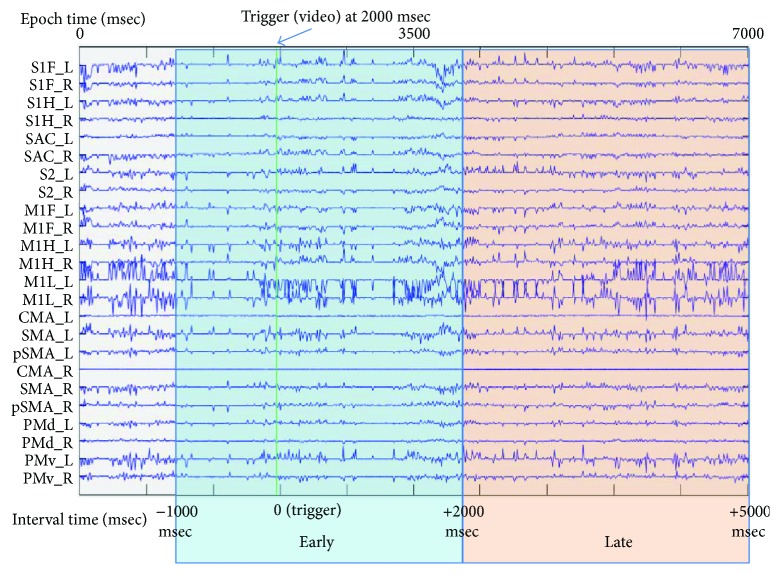
Activation time series of all regions of interest (ROIs) of the sensorimotor cortex during a random epoch and definition of time intervals around the trigger (onset of the video presenting the motor imagery task).

All signal preprocessing was performed using a custom script on the FieldTrip toolbox for MATLAB [49]. Raw data from those selected channels was band-pass filtered at 0.5–30 Hz using a zero-phase FIR filter and subsequently downsampled at 100 Hz. We visually examined continuous EEG signal time series of each subject to detect bad electrodes that showed large drifts from their mean value and then removed these electrodes. Epochs were then initially extracted from −2000 msec to +5000 msec centered on the trigger (visual cue). Subsequently, independent component analysis was performed on the concatenated continuous data (of each session) using the second-order blind identification method [50]. Independent components corresponding to eye blinks and muscle artifacts were identified and removed from the epoched data. Bad electrodes were then interpolated using spherical splines interpolation [51]. Finally, the epoched data were split into two time intervals (Figure 3), which will be referred to as “early” (early onset imagery from −1000 msec to +2000 msec around the trigger) and “late” (late continuous imagery from +2000 msec to +5000 msec after the trigger). While shorter time windows have also been used in similar analyses [52], differences in the behavior of alpha and beta rhythms between the window around the imagery onset and later windows have been identified with regard to networks [53], relative power [54], and event-related desynchronization [55]. The data from one subject (from the healthy group) was exempted from further analysis, as this preprocessing methodology did not result in sufficiently clean epoched data.

#### 2.2.2. Current Cortical Density

The solution of the forward problem, the lead field matrix that best describes the conduction from the current cortical density (CCD) source model (Table 3) to scalp potentials, is based on the following equation:

(1) $$\begin{array}{c} m=Ld+b, \end{array}$$

where *m* refers to the M simultaneous electrode voltage recordings, *d* refers to the N current dipoles in the current cortical density model, *b* is the noise vector, and *L* is the abovementioned lead field matrix [56]. We used the solution applied in eConnectome toolbox for MATLAB [57, 58] of the forward problem which is a high-resolution lead field matrix relating 2054 scalp triangles to 7850 cortical dipoles. The lead field matrix is derived using a three-layer block element modifier model based on the Colin27 Montreal Neurological Institute brain [59]. The dipoles were constrained to the gray matter with orientations perpendicular to the local cortical surface, under the assumption that the primary source of measured EEG signal is local groups of pyramidal neurons of the cortex firing synchronously and is arranged perpendicular to its surface [60]. In our case, a subset of the lead field matrix was used for the 64 selected EEG electrodes.

Weighted minimum norm estimate was used to solve the ill-posed inverse problem (Table 3) by minimizing the source space energy based on the fact that the power of the source dipoles is limited by the cortex physiology [61]. Minimum norm estimate aims at minimizing the following equation:

(2) $$\begin{array}{c} J\left(d\right)={\left\|m-Ld\right\|}^{2}+\lambda {\left\|d\right\|}^{2}, \end{array}$$

where *m* refers to the actual recordings from the scalp, *d* is the simultaneous current dipoles to be calculated, *L* is the lead field matrix, *λ* is the regularization parameter, and |*d*|^2^ is the regularization term which in our case refers to the energy of the solution's dipoles. The first term in the above equation represents the error between the actual and predicted electrode recordings. The second term is the penalization term, which aims at enforcing the abovementioned energy restriction. Lastly, *λ*, which balances the effect of the penalization term, was calculated using the L-curve method [62]. The solution of minimum norm estimate was derived using Tikhonov regularization in the regularization toolbox [63]. 24 custom-defined ROIs were created at the surface of the cortex model, in order to proceed to connectivity analysis, as illustrated in Figure 2. The ROI time series signal (Figure 3) was calculated by averaging the amplitude from all included cortical current dipoles.

#### 2.2.3. Functional Connectivity

In total, 24 ROIs were defined on the cortical source model, consisting of the following areas bilaterally: presupplementary motor area (pSMA), supplementary motor area (SMA), dorsal premotor area (PMd), ventral premotor area (PMv), cingulate motor area (CMA), primary foot motor area (M1F), primary hand motor area (M1H), primary lip motor area (M1L), primary foot somatosensory area (S1F), primary hand somatosensory area (S1H), secondary somatosensory area (S2), and somatosensory association area (SAC) (Figure 2). Their average activation time series were computed for every time interval.

Directed transfer function (DTF) [64] was used (Table 3) in order to calculate functional cortical connectivity of the sensorimotor network consisting of the 24 ROIs as nodes [44], computing causal relations among the nodal activation time series. The produced connectivity matrices were thresholded, using the surrogate data method with testing of significance of connections, instead of using absolute or relative thresholding [65–67]. During computation of DTF, a number of 1000 surrogate permutations and a significance level of 0.05 were set, resulting in partially connected matrices with only the most significant causal connections. DTF is a measure based on Granger causality [68] that uses the multivariate autoregressive model described by the following function:

(3) $$\begin{array}{c} X\left(t\right)=\overset{p}{\underset{j=1}{\sum }}A\left(j\right)X\left(t-j\right)+E\left(t\right), \end{array}$$

where *p* is the model order, *X*(*t*) contains the ROIs values at time *t*, *E*(*t*) is the residual noise vector, and *A* is a coefficient k × k-sized matrix [44]. Using the above equation, the *A* matrices are computed by means of the minimalization of the residual noise *E*.

The order of the multivariate autoregressive model [69] was chosen to be 8 after considering the following criterions [70]: (a) tests that demonstrated an optimal order of 10 for a sampling rate of 128 Hz for modeling EEG spectra [71, 72]; (b) the model order should be smaller than *τ* × Fs, where *τ* is the expected lag between two brain processes and Fs the sampling rate; (c) better to err on the side of selecting a larger model order, (d) using the same model order for all DTF computations.

Equation (3) is described in the frequency domain as

(4) $$\begin{array}{c} E\left(f\right)=A\left(f\right)X\left(f\right),\\ X\left(f\right)=A^{-1}\left(f\right)E\left(f\right)=H\left(f\right)E\left(f\right), \end{array}$$

where *H*(*f*) is a transfer matrix of the system, and it contains information about the relationships between signals. It is nonsymmetric, so it allows for finding causal dependencies. DTF is then computed by the equation:

(5) $$\begin{array}{c} {DTF}_{j\to i}^{2}\left(f\right)=\frac{{\left\|H_{ij}\left(f\right)\right\|}^{2}}{\sum_{m=1}^{k}{\left\|H_{im}\left(f\right)\right\|}^{2}}. \end{array}$$

DTF describes casual influence of channel *j* on channel *i* at frequency *f*. For our analysis, DTF was computed for the networks formed at the frequency bands of alpha rhythm at 8–12 Hz (“alpha networks”) and beta rhythm at 13–30 Hz (“beta networks”), as those are considered the brainwaves most relevant to the sensorimotor processes [73].

#### 2.2.4. Network Analysis

Network analysis in terms of descriptors of the weighted directed graphs (“network properties”) was performed with the brain connectivity toolbox for MATLAB [74] for the *alpha* and *beta* networks formed during *early* and *late* time intervals. Out-strength (OS), in-strength (IS), and clustering coefficient were computed for each of the 24 nodes of the network during each task of imagery. Characteristic path length (CPL), mean clustering coefficient (CC), and density (*D*) were also computed at the level of graphs of each task of imagery. Topology of small-worldness (SW) was then derived from these network properties for each task of imagery [44]. To facilitate further analysis, all properties were averaged for the 8 imagery categories mentioned in Experimental Procedure. A summary of the interpretation of these network properties is also presented in Table 3.

CPL, a measure of integration, calculates the sum of the shortest distances among connected graph nodes, divided by the number of nodes [75, 76]. It is described by (6), where *Li* is the average distance between node *i*  and the other node, and *d*_*ij*_ is the shortest path between nodes *i* and *j*. The distance matrix was computed using the logarithmic conversion of weights by the Floyd-Warshall algorithm [77], as implemented in the BCT [78].

(6) $$\begin{array}{c} CPL=\frac{1}{n}\sum_{i\in N}Li=\frac{1}{n}\sum_{i\in N}\frac{\sum_{j\in N,j\neq i}d_{ij}}{n-1}. \end{array}$$

Global CC, a measure of segregation, estimates the tendency of the graph nodes to organize into clusters [79, 80]. It is described by (7), where *Ci* is the clustering value for a node *i*, *k*_*i*_ is the node's degree, and *t*_*i*_ is the number of neighboring nodes that connect to each other in triangles around node *i*.

(7) $$\begin{array}{c} CC=\frac{1}{n}\sum_{i\in N}Ci=\frac{1}{n}\sum_{i\in N}\frac{2t_{i}}{k_{i}\left(k_{i}-1\right)}. \end{array}$$

*D* is the ratio of a network's actual connections to the maximum possible connections. In our example, the graphs were only partially connected, and the connection weights were ignored, since all connections were considered significant as computed by DTF with testing for statistical significance (*p* = 0.05) by surrogate data. It is therefore described by (8), where *E* is the ensemble of the network's connections, and *V* is the ensemble of the network's nodes.

(8) $$\begin{array}{c} D=\frac{\left|E\right|}{\left|V\right|\left(\left|V\right|-1\right)}. \end{array}$$

SW is defined as the combination of short paths and high clustering in a network, when compared to random networks with comparable paths constructed by the same number of nodes and connections. This property has commanded attention as an important brain network characteristic that models the brain's effective communication patterns [81–83]. It is described by (9), and in our study, the comparison of CPL and CC was made against 10,000 random networks. These random networks were directed graphs, with the same number of nodes and edges as the original, and they were generated using the brain connectivity toolbox [84]. CPL and CC were computed for each random network and compared against the original, in a process that was iterated 10,000 times to produce a range of SW values. The range of values was then averaged to produce a single robust value of the SW property for each original network.

(9) $$\begin{array}{c} SW=\frac{CC/{CC}_{\mathrm{rand}}}{CPL/{CPL}_{\mathrm{rand}}}. \end{array}$$

The strength of node strength is the sum of the weights of connections to or from that node. The IS, therefore, is the sum of incoming connection weights, and the OS is the sum of outgoing connection weights [74]. They are described by (10) and (11), respectively, where *C*_*ij*_ is the weighted directed *N* ^∗^ *M* connectivity matrix, with a direction of *i* → *j*, *N* is the number of columns, and *M* is the number of rows.

(10) $$\begin{array}{c} \text{IS}=\overset{N}{\underset{j=1}{\sum }}C_{ij}, \end{array}$$

(11) $$\begin{array}{c} OS=\overset{M}{\underset{i=1}{\sum }}C_{ij}. \end{array}$$

### 2.3. Statistical Analysis

Adjacency matrices computed by directed transfer function were compared between healthy and patient groups using the network-based statistic toolbox for MATLAB [85]. We performed the statistical analysis using the false discovery rate on the general linear model with *t*-test [86], a significance level of 0.05 and 50,000 permutations. Using a between-group design, we compared alpha and beta networks of healthy subjects to alpha and beta networks of SCI subjects for each imagery category, elaborating the comparisons for the effect of early and late intervals. We also compared alpha to beta networks of each category and time interval using a within-group design. Differences in networks were visualized using the BrainNet Viewer for MATLAB [87].

Statistical analysis of age, imagery capacity, and computed network properties was performed in IBM SPSS Statistics (version 23), and we set a significance level of 0.05 for all statistical tests. All variables were explored for normality assumption (healthy and SCI groups as grouping factor) using visual inspection of histograms, normal Q-Q plots and boxplots, skewness and kurtosis [88–90], and normality tests (Shapiro-Wilk test and Kolmogorov-Smirnov test) [91, 92]. Depending on normality assumption, different analyses were performed (paired *t*-tests or Wilcoxon signed-rank tests). Normality assumption was met for the variable age and for the VVIQ score for both groups. Independent sample *t*-tests were performed to reveal significant age and VVIQ differences between the two groups. As the groups did not differ either for age distributions (healthy-skewness: 0.407 (SE = 0.687), kurtosis: −1.418 (SE = 1.334); SCI-skewness: 0.651 (SE = 0.687), kurtosis: −0.752 (SE = 1.334)) or for their reported imagery capacity (VVIQ: *t* = −1.094, df = 8, and *p* = 0.306), the rest of the statistical analysis was planned accordingly.

We planned within-group comparisons of brain network properties using as grouping factor the rhythm (alpha, beta). Differences of variables between the two rhythms were calculated for the categories of visual motor imagery tasks, separately at early and late time intervals. Subsequently, we calculated within-group comparisons of brain network properties using as grouping factor the time interval (early, late). Between-group comparisons were performed using the calculated differences of variables at the two time intervals with either independent samples *t*-tests or Mann–Whitney *U* tests.

Nodal strengths, both incoming and outgoing, were averaged across different motor imagery tasks, rhythms, and time intervals, and total nodal strengths were calculated. They were tested for normality assumption for both groups and analyzed within groups using descriptive statistics and between groups (SCI, healthy) using Mann–Whitney *U* test. Targeted differences between nodes CMA_L and CMA_R were tested for statistical significance using either Pearson or Spearman correlation coefficient depending on normality assumption.

## 3. Results

### 3.1. Functional Connectivity

**Figure 4:**
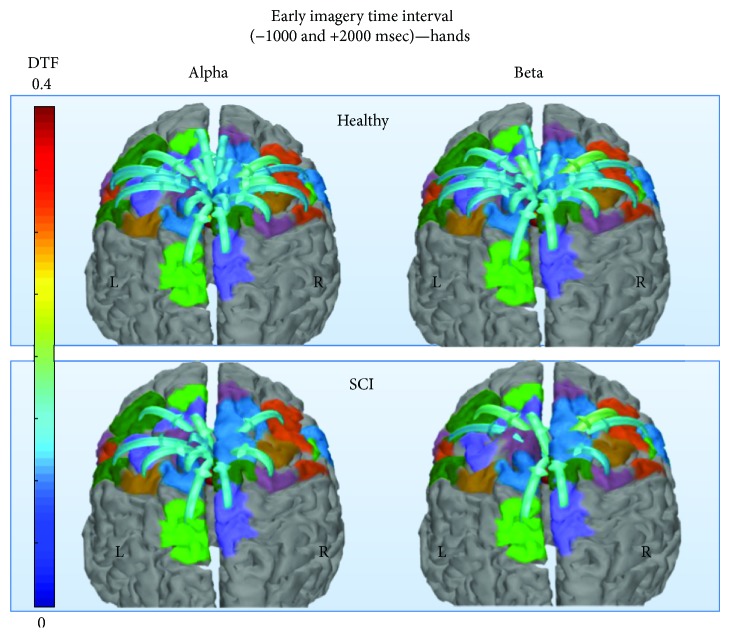
Average information transfer (calculated by directed transfer function) of healthy and SCI groups calculated for alpha (left) and beta (right) rhythm networks during the early imagery time interval, for the hands motor imagery category. Connections between the bilateral cingulate motor areas (CMA_R ← → CMA_L) presented the highest information transfer. Only connections with at least 25% of max information transfer among all statistically significant connections are displayed.

Visualizations of connectivity maps for the two groups (SCI, healthy) were made using the eConnectome toolbox for alpha and beta networks during both time intervals averaged across the motor imagery categories (Figure 4). The highest information transfer in all examined networks came from the bilateral cingulate motor areas including their reciprocal communication. In both groups, the maximum incoming nodal strength was observed in right CMA (Table 4), whereas the maximum outgoing nodal strength was found in left CMA (Table 5). Between-group comparisons revealed significant differences in total nodal strengths, both incoming and outgoing.

**Figure 5:**
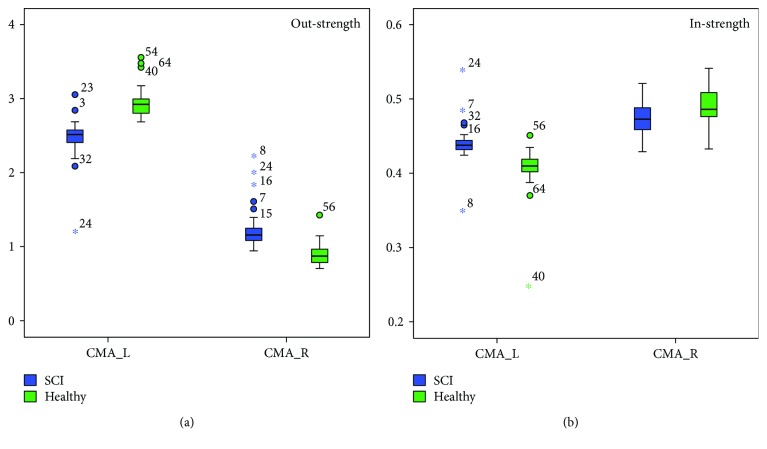
Nodal strengths (a: out-strength, b: in-strength) of bilateral cingulate motor areas for both subject groups. Left cingulate motor area (CMA_L) showed the highest out-strength and right cingulate motor area (CMA_R) showed the highest in-strength in the network. SCI subjects presented significantly higher CMA_R out-strength and CMA_L in-strength than healthy subjects. “∗” represent extreme values and “o” represent outliers.

More precisely, significant differences in incoming strengths were found bilaterally in CMA, SMA, S1H, PMd, and PMv as well as in the left S1F. Healthy participants showed higher incoming strengths in all aforementioned nodes apart from the left CMA (Figure 5) compared to the SCI participants (Table 4).

Outgoing strengths were found to be significantly different between groups in all nodes (Table 5) apart from the right S1F. In more detail, SCI group showed considerably higher outgoing strengths in the S1F and SAC in the left hemisphere, in S1H and CMA in the right hemisphere as well as in S2, PMd and PMv bilaterally. In the remaining nodes, healthy participants were found to have increased outgoing strengths compared to SCI participants.

**Figure 6:**
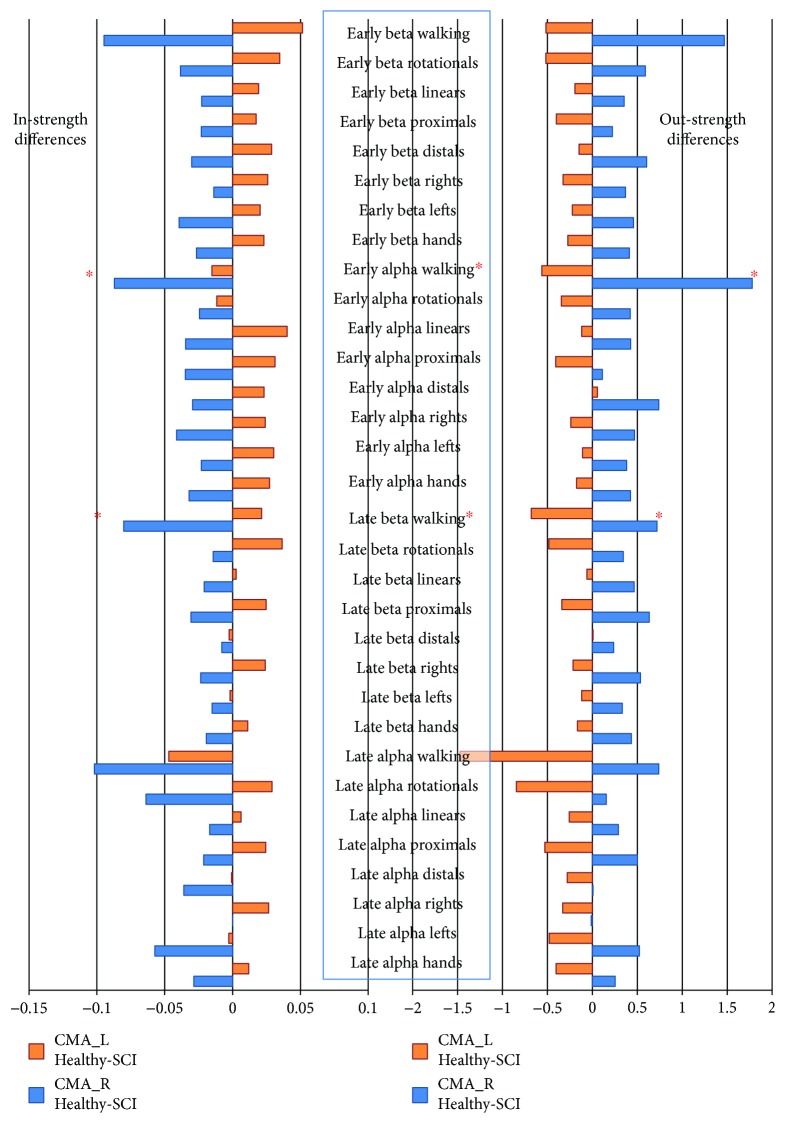
Differences of group averages (healthy-SCI) of in-strength (IS) and out-strength (OS) of cingulate motor areas (CMAs) during all motor imagery categories. A trend was revealed, in which OS of CMA_R was consistently higher in the healthy than the SCI group. OS of CMA_L was consistently lower in the healthy than the SCI group. The opposite held true for IS of those nodes. This trend reached statistical significance only for early alpha walking (*p* = 0.002) and late beta walking (*p* = 0.006) tasks.

When calculating differences of group averages (healthy group-SCI group) of in-strength (IS) and out-strength (OS) of cingulate motor areas (CMAs) during all motor imagery categories, a possible trend was revealed. OS of CMA_R was consistently higher in the healthy group, while OS of CMA_L was consistently higher in the SCI group. The opposite held true for IS of those nodes (Figure 6). Between-group differences in CMA_R were negatively correlated to those in CMA_L in targeted imagery categories (early alpha walking (*r* = −0.867, *p* = 0.002), late alpha walking (*r* = −0.250*p* = 0.517), early beta walking (*r* = −0.502, *p* = 0.169), and late beta walking (*r* = −0.827, *p* = 0.006)).

### 3.2. Network-Based Statistics between Groups and within Groups

**Figure 7:**
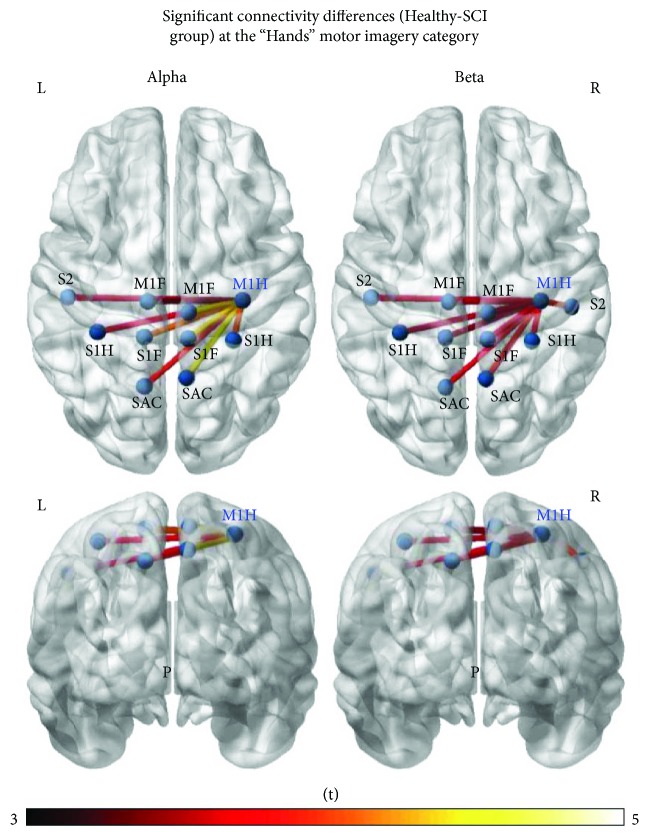
From the comparison of the networks of healthy and SCI subjects, a subset of network connections emerged as significantly stronger in the healthy group than in the SCI group for both the alpha and beta networks of “hands” motor imagery category, as calculated by network-based statistics—false discovery rate methodology.

Important differences of connectivity were found only in between groups (SCI against healthy), where a subset of connections had significantly higher FC in the healthy group than in the SCI group in the hands motor imagery category (Figure 7). This subset included connections with lower FC in the SCI group of M1H_R to bilateral primary foot motor areas (M1F), primary foot and hand sensory areas (S1H, S1F), the somatosensory association areas (SAC), and the secondary sensory areas (S2). This finding persisted in both alpha and beta networks when testing with *t*-test.

When we further tested the networks of our participants by grouping the SCI subjects by outcome (positive and negative), no differences were found between the networks of the two groups. During all permutations, the *p* value of the false discovery rate did not approach statistical significance (*p* > 0.05). Also, testing for other imagery categories did not reveal significant differences, with the exception of the walking category. Comparing the networks of healthy and patients during the walking imagery category, significantly greater S2_L-PMv_R connectivity was found in the SCI group.

Furthermore, when testing for main effect of within time interval and brainwave rhythm within the healthy and patient groups, no further statistical significant differences of the connectivity weights of the network were observed.

### 3.3. Analysis of Network Properties

#### 3.3.1. Within-Group Comparisons of Graph Properties between Alpha and Beta Showed Less Segregation, Less Integration, Greater Density, and Less Effectiveness of Beta Networks

When exploring within-group differences of graph properties using as grouping factor the rhythm (alpha, beta), beta networks showed *less segregation*, *less integration*, and *less overall effectiveness* compared to alpha networks. CPL, CC, and SW showed significantly lower values in beta compared to alpha networks, *in both early and late time intervals*. These findings were observed during nearly all imagery categories in both SCI and healthy group.

**Figure 8:**
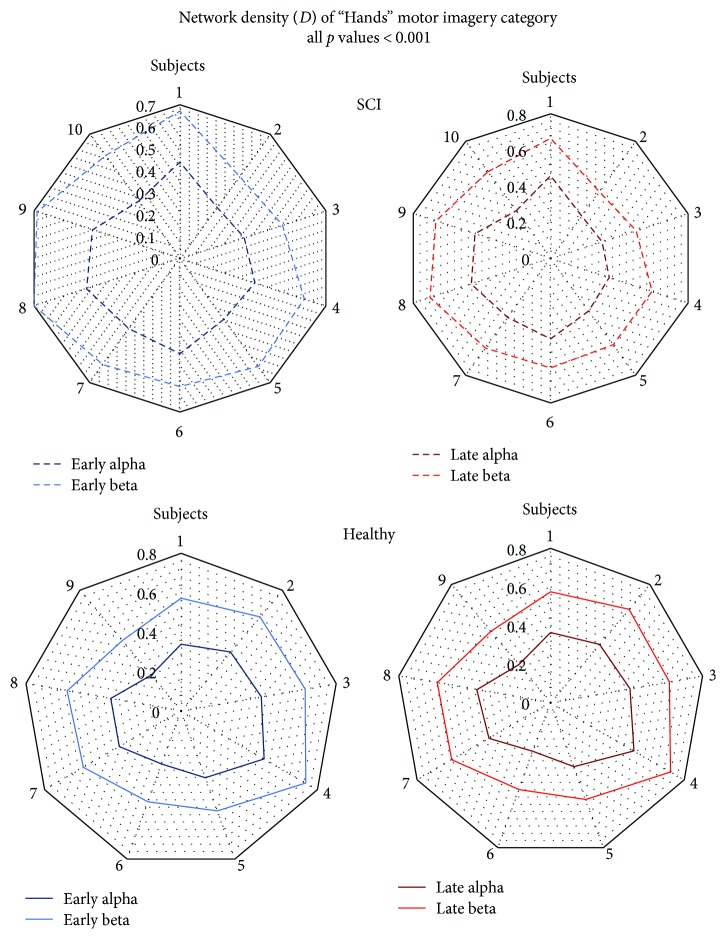
Network density was significantly higher in beta than alpha networks (all *p* values < 0.001) of the “hands” motor imagery category during both early and late time intervals for all subjects of both the SCI and the healthy group (all *p* values < 0.001). This finding was also consistent across every other motor imagery categories in both groups (all *p* values < 0.001).

On the opposite, beta networks showed *greater density* compared to alpha networks. *D* was significantly greater in beta networks in both early and late time intervals of all imagery categories in both SCI and healthy group (Figure 8). Aggregated statistical test results and *p* values for abovementioned findings can be found in supplementary material (available here). Specific exemptions are detailed below, as differences of graph properties in beta network compared to alpha did not reach statistical significance only in walking category, in the following cases: (a) in SCI group, CPL during the late interval, (b) in SCI group, CC and SW during the early interval, and (c) in healthy group, CC during the late interval.

#### 3.3.2. Within-Group Comparisons of Graph Properties between Early and Late Time Intervals Showed (1) Less SCI Network Integration during Late Walking Imagery, (2) Greater SCI Network Segregation and Stable Effectiveness for Distal Tasks during Late Imagery, and (3) Less Healthy Network Segregation and Effectiveness for Distal Tasks during Late Imagery

When exploring within-group differences of graph properties using as grouping factor the time interval (early, late), few significant differences were observed.

Regarding network integration, significant difference of CPL values was shown only SCI group's alpha networks during the *walking* task (*t* = 2.743, df = 9, and *p* = 0.023). More precisely, SCI subjects were characterized by lower path lengths at the second stage of the task (late) (early alpha walking CPL: 7.809; late alpha walking CPL: 7.032). Changes in the CPL in the beta rhythm comparing the two time intervals were not observed. Also, no significant difference was found for healthy subjects.

Regarding network segregation, significantly higher CC values in the SCI group were observed in the alpha band of *distal* imagery category (*t* = −2.574, df = 9, and *p* = 0.030; early alpha distal CC: 0.0076; late alpha distal CC: 0.0082). Considerable differences in mean CC at the beta band were not found. In healthy participants, considerably lower CC value was found only in alpha band of the *left* category (*t* = 2.435, df = 8, and *p* = 0.041; early alpha left CC: 0.0094; late alpha left CC: 0.0086).

Regarding network density, healthy group showed significantly higher *D* values at the late stage of *linear* imagery tasks in alpha rhythm (*t* = −2.543, df = 8, and *p* = 0.035; early alpha linear *D*: 0.3595; late alpha linear *D*: 0.3713), whereas density was considerably less at the late stage of *proximal* tasks in beta rhythm (*t* = 3.038, df = 8, and *p* = 0.016; early beta proximal *D*: 0.5904; late beta proximal *D*: 0.5784). SCI group showed greater density when comparing the two time intervals of *right* imagery category in alpha band (*t* = −2.962, df = 9, and *p* = 0.016; early alpha right *D*: 0.3663; late alpha right *D*: 0.3801), but no alterations were found in beta networks.

Regarding overall network effectiveness, significant results of SW were found for healthy subjects at *distal* imagery tasks in both alpha and beta rhythms (alpha: *t* = 2.201, df = 8, and *p* = 0.059; beta: *t* = 3.044, df = 8, and *p* = 0.016). In more detail, significantly lower values of SW were observed in distal imagery tasks between the two time intervals (early alpha distal SW: 1.553; late alpha distal SW: 1.406; early beta distal SW: 1.159; late beta distal SW: 1.137). For the SCI group, considerable differences were not observed.

#### 3.3.3. Between-Group Comparisons of Graph Properties Showed Not Only Similar Network Integration and Density But Also Greater Segregation and Effectiveness of Alpha Band Networks in Some Imagery Categories for the Patients

When exploring between-group differences of graph properties, few significant differences were observed. Comparisons of CPL and *D* did not reveal any considerable difference across any imagery category (supplementary material), showing similar network integration and network density of the networks of healthy and patient subjects.

Regarding network segregation, significant changes in CC were observed at the *left* imagery tasks (*t* = 2.672, df = 17, and *p* = 0.016), at the *rotational* imagery tasks (*t* = 2.104, df = 17, and *p* = 0.051), and at *distal* imagery tasks (*U* = 20.00, *p* = 0.041), all appearing in alpha band. In more detail, SCI subjects seem to show greater CC of alpha networks in the late part of the aforementioned imagery tasks (alpha left dif (mean)—SCI: 0.00019, healthy: −0.00084; alpha distal dif (median)—SCI: 0.00049, healthy: −0.00075; alpha rotational dif (mean)—SCI: 0.00061, healthy: −0.0010). Significant differences were not found between groups in the beta networks of all tasks.

Regarding overall network effectiveness, the SCI group seems to have only a significant change in mean SW of alpha network while performing VMI on *distal* imagery tasks compared to healthy (*t* = 2.365, df = 17, and *p* = 0.030). More precisely, SCI group seems to show greater SW of alpha networks in the late part of the distal tasks (alpha distal dif (mean)—SCI: 0.0253, healthy: −0.1467). Other between-group differences were not observed (supplementary material).

## 4. Discussion

### 4.1. Functional Connectivity

A subset of the sensorimotor network during hands motor imagery was shown to have significantly lower functional connectivity power in the SCI group compared to the healthy group, a finding from analyses of the general linear model. This subset included connections of the M1H_R cortical area (theoretically the nondominant hand primary motor area for right-handed subjects) with other motor and sensory cortical areas. This subset also excluded the “assistive” motor nodes (CMA/SMA/pSMA/PMv/PMd). Interestingly, among these excluded nodes were also the ones that were shown to have consistently higher OS and IS, for all imagery categories, as we will discuss later on. The subset of connected nodes to M1H_R included bilateral primary foot motor areas (M1F), primary foot and hand sensory areas (S1H, S1F), the somatosensory association areas (SAC) located in superior parietal cortex (SPC), and the secondary sensory areas (S2). Small differences between alpha and beta rhythm networks can be observed, whereas most of those connections' lower FC reached statistical significance for either rhythm. These cortical areas can be identified as the point of origin of the pyramidal tract and the point of conclusion of the major somatosensory tracts. This finding could suggest that chronic disruption of reciprocal communication between the brain and spinal cord, even in noncomplete injuries, could result in permanent significant decrease of connectivity between a subset of the functional sensorimotor network at the cortical level. This effect was observed regardless of positive or negative neurological outcome since grouping SCI subjects by outcome did not reveal any differences regarding this finding. While the lack of difference between those two clinically and functionally different subgroups of patients could be affected by a lack of statistical power when comparing small samples, a possible explanation could also be explored along the lines of mental imagery capacity. Motor execution and motor imagery do present differences in the level of neural networks that are affected by the subjects' quality and intensity of imagination [93]. In our study, the patients did not report differences in vividness of visual imagery to that of the healthy participants, as measured by the VVIQ questionnaire at the time of the experimental procedure. A degree of “though extinction process” has been reported in chronic paralysis [94], but since this is not the case in our investigation, it is possible that the lack of difference in the networks of patients with positive and negative outcomes could be due to the unaffected mental capacity of the participants.

Further significant differences between the healthy and patient groups were found with regard to nodal strengths. Almost all nodes had significantly different out-strength between the two groups. An interesting pattern can be identified. Primary motor areas, supplementary motor areas, left presupplementary motor area, and left cingulate motor area show significantly higher out-strength in the healthy group. On the contrary, premotor areas, right presupplementary motor area, and right cingulate motor area show significantly higher out-strength in the SCI group. Incoming connection strengths to primary somatosensory areas, premotor areas, supplementary motor areas, and right cingulate motor area are higher for healthy participants, while in-strength of left cingulate motor area is higher for SCI participants. Despite the differences in strengths of those nodes, similar patterns of connectivity were found for both groups [12, 13]. The significantly reduced nodal strengths could reflect the disconnection itself and the reduced input and output of the sensorimotor pathways in spinal cord injury. Nonetheless, the higher in-strengths of left cingulate motor area and out-strengths of premotor areas and right pSMA and CMA in SCI group could indicate an attempt of the sensorimotor network to compensate for the impaired function [10, 11, 95].

CMA areas have been previously identified as important information hubs for sensorimotor networks, especially those of beta rhythm [9, 11]. In our study, this attribute is confirmed, since bilateral CMA areas consistently received the greater inflow and contributed the greater outflow in terms of connection strengths for all categories of motor imagery. Moreover, their reciprocal communication constituted the most powerful connections of every examined network. On the other hand, an important role has been identified for the SMAs [11–13] that have been shown to present notable outflow during motor imagery tasks and form clusters with the CMAs. Their role was asserted in our work previously too [44, 96], but it is not so apparent in our current study, where the SMAs were not among the top contributors in either outflow or inflow. Although not easily explained, the meaning of this finding can be explored along possible factors: (a) the random-oddball (unexpected imagery task) paradigm of presentation, (b) the MVAR model order set, and (c) the definitions of the midline network nodes themselves. The degree that each factor possibly contributed to this finding is an issue for further investigation. An example of SMAs and primary motor areas not presenting the greatest strength during hands motor imagery has also been recently reported in a study [97] where the authors used transcranial direct current stimulation to affect the connectivity of a broader definition of sensorimotor ROIs.

With regard to differentiating different upper limb motor imagery tasks, our results did not produce significant differences in terms of spatial patterns specific to certain tasks. Moreover, network-based differences between healthy and patients, although significant for the all-inclusive upper limb imagery category, did not reach statistical significance for specific categories, suggesting possibly a lack of statistical power for these categorical differences. This is not unexpected, since connectivity features, in general, have so far shown only moderate success in classification of motor imagery tasks [98, 99]. It should be also noted that some effort has been made in analyzing effective networks of compound motor imagery tasks [100]. Differentiating anatomical levels and consecutive classification should perhaps be better explored along the lines of time-varying connectivity [95, 101–103] instead of spatial pattern analysis.

Walking motor imagery, while it also did not reveal specific connectivity patterns, produced the most promising results in terms of classification, in accordance to previous studies suggesting that maximally different conditions should be explored [98]. Walking motor imagery category was the only one where the negative correlation between-group differences of the two cingulate motor area strengths reach statistical significance in half of the studied cases, those of early alpha rhythm walking networks (*p* = 0.002) and of late beta walking networks (*p* = 0.006). Moreover, the comparison of networks of healthy and patient subjects produced at least one significantly stronger connection, between the right ventral premotor area and the left secondary somatosensory area, although it is unclear whether this can be attributed to plasticity or merely to SCI-induced disconnection sequelae. To the best of the authors' knowledge, this is the first electroencephalographic study of functional cortical connectivity after incomplete spinal cord injury, and it is also the first functional cortical connectivity study examining multiple motor imagery tasks in those patients regardless of recording modality.

### 4.2. Analysis of Network Properties

Analysis of within-group effect of rhythm produced the most consistent results. According to the revealed pattern, alpha networks present lower integration (as measured by CPL), higher segregation (as measured by CC), while being less dense and more “effective” (as measured by SW) than beta networks. These findings are present across all motor imagery categories and they closely match findings from our previous study on the role of alpha and beta rhythms in sensorimotor networks [44, 104]. Our previous work suggested a pattern where alpha rhythm engaged local information processing using greater wiring costs [105], and that beta rhythm assumes a coordinative role during the sensorimotor process [106]. These findings were then observed on different ROI models and during simpler but far more repetitive motor imagery and motor execution tasks. They are also now replicated on a wider definition of the model of ROIs and during multiple, more complex, random motor imagery tasks. More importantly, these findings have now also been confirmed on networks of SCI patients with incomplete injury, allowing us to attempt to model the behavior of other between-group and within-group findings based on this pattern of alpha and beta organization.

Between the two groups, the fact that CPL and *D* were not significantly different, neither in alpha nor in beta networks, allows us to make direct comparisons of their sensorimotor network organization since they reach the same level of wiring costs and node integration. Moreover, the few between-group differences were observed mostly in alpha rhythm, which could be interpreted as differences only in local processing in the sensorimotor network of SCI patients. Increased functional segregation (CC in left, rotational and distal categories) and increased “effectiveness” of the network (SW in distal) were found for certain categories of motor imagery. More importantly, they were observed for *distal* arm imagery tasks, those that correspond to spinal cord levels below the level of injury, as the majority of the SCI subjects included in our study suffered from mid to low cervical SCI (C4–C8). As those differences were also observed during the late time interval, they can possibly be interpreted as an effect for delayed adaptation (compensation) of the sensorimotor network at the cortical level. This could possibly fall in line with reported increased network fault tolerance [10] and an increase of local efficiency and communication between closest cortical areas [15] during paralyzed foot motor imagery that has been reported in chronic complete SCI subjects.

Regarding the walking imagery category in our study, walking networks were the only where alpha and beta rhythm differences did not reach statistical significance in certain cases. Indeed, this previously reported increase of local efficiency and close communication in complete SCI appears to also be possible in incomplete injuries as well. The walking beta networks did not show less segregation and effectiveness than the walking alpha networks of incomplete SCI subjects during the early imagery part and also did not show less integration during the late imagery part, as was the case with the upper limb imagery categories. These findings suggest the presence of a phenomenon that has been attributed to adaptive plasticity and compensation when it regards patients with complete injuries [10, 15]. Within-group effect of time interval for upper limb motor imagery categories was far more sporadic, showing greater network segregation and less network integration of the alpha rhythm network in the patient group during the late imagery in some categories. What could interestingly fall into place with the rest of the interpretation is that healthy subjects display a drop of overall network effectiveness (as depicted by SW) in both alpha and beta networks during the late imagery part. In the SCI group, such a difference was not observed, an observation that could possibly be attributed to the same delayed compensatory effect induced by the injury. Since indeed the rest of the observed effects are not consistent, our reported findings cannot be obligatory attributed to neuroplasticity effects. Therefore, it is evident that more investigation in the direction of modeling the effect of spinal cord injury on the effective connectivity of the brain during different time points and injury severity conditions is needed before drawing accurate conclusions.

### 4.3. Limitations and Future Work

Among limitations of our study, one investigating functional connectivity should remain wary when the analysis reveals significant differences between the groups, as those differences are usually very small and not always clear if functionally or clinically meaningful. As such, it remains difficult to identify compelling advantages of graph theory-based analysis of brain activity over other approaches to provide additional important insight into the effects of SCI on brain activity. Functional connectivity at the source level also suffers from certain disadvantages including localization error, smoothing effect, and a degree of uncertainty of the connectivity between spatially close nodes [107]. There are several factors contributing to source localization errors, induced most importantly by the forward model but also by the inverse. The resolution of the source reconstruction is determined by the source space, with 4.69 mm of average source spacing and 22.04 mm^2^ average surface area per source. Nonetheless, resolution of the source space is considered sufficient for the purpose of the study, and the same model has been previously used by similar studies [9–15]. The 3-layer block element modifier forward model introduces errors through the use of a general template anatomy, modeling of skull conductivity as isotropic, not modeling cerebrospinal fluid and conductivity ratios. Moreover, it is known that EEG boasts great temporal but suffers from low spatial resolution and has been traditionally considered able to detect rapid brain dynamics in a trade-off with source estimation and low signal to noise ratio due to volume conduction effect [108, 109]. EEG in general greatly suffers from anisotropic conductivity of skull leading to signals blurring and to low EEG spatial resolution. In accordance, the localization error of deeper sources is considered greater than swallower ones. An interesting approach to address these problems would be the investigation of time-adaptive connectivity with a focus on temporal alterations of important connections rather than spatial [64], while using individual subject anatomy, an approach that we aim to explore in our future work.

## 5. Conclusions

We observed that chronic disruption of reciprocal communication between the brain and spinal cord, even in the context of incomplete injuries, could result in permanent significant decrease of connectivity between a subset of the functional sensorimotor network at the cortical level. This effect was observed regardless of positive or negative neurological outcome since grouping SCI subjects by outcome did not reveal any further difference. Cingulate motor areas were identified as important information hubs in different categories of motor imagery as they consistently showed the highest in-strengths (CMA_L) and out-strengths (CMA_R) in both groups of participants. While SCI subjects also followed the same pattern, they had higher outflow from left CMA and higher inflow to right CMA than healthy subjects. For both groups, alpha networks were less dense while having both longer average paths and more clustering than beta networks in almost all imagery categories. SCI patients showed signs of increased local processing in the late part of imagery, possibly an adaptive compensatory mechanism of injury-induced neuroplasticity.

## Figures and Tables

**(a):** 

SCI group	Age	Gender	Cause	AIS	NLI	VVIQ
CSI-02-001	28	f	MVA	C	C4	54
CSI-02-002	52	m	MVA	D	C4	69
CSI-02-003	42	m	MVA	D	C8	68
CSI-02-004	70	m	Fall	D	C5	76
CSI-02-005	60	m	Fall	E	C6	70
CSI-02-006	28	m	MVA	D	C5	56
CSI-02-007	30	m	MVA	E	C5	67
CSI-03-001	47	m	Fall	A	T7	72
CSI-03-002	29	f	MVA	B	T4	60
CSI-03-003	74	m	Other	B	T4	65
*Mean (SD)*	*46.00 (17.64)*					*65.70 (7.04)*

**(b):** 

Healthy group	Age	Gender	VVIQ
CSI-04-001	27	f	77
CSI-04-007	51	m	75
CSI-04-003	43	m	56
CSI-04-006	71	m	70
CSI-04-009	63	m	70
CSI-04-004	28	m	46
CSI-04-005	31	m	58
CSI-04-008	47	m	80
CSI-04-002	27	f	75
CSI-04-010	74	m	63
*Mean (SD)*	*46.20 (18.27)*		*67.00 (10.09)*

**Table 2:** Presented motor tasks for one upper extremity (left or right): 16 motor tasks were presented (8 independent movements (degrees of freedom) ^∗^ 2 directions of movement) and were then classified by proximity (proximal or distal tasks) and resulting motion (linear or rotational). For both upper extremities, the subjects watched and performed visual imagery of 32 motor tasks in total.

Independent movement	Direction	Proximal/distal	Linear/rotational
Shoulder	Arm down	Proximal	Linear
Shoulder	Arm up	Proximal	Linear
Shoulder	Arm left	Proximal	Linear
Shoulder	Arm right	Proximal	Linear
Elbow	Forearm down	Proximal	Linear
Elbow	Forearm up	Proximal	Linear
Forearm	External rotation	Proximal	Rotational
Forearm	Internal rotation	Proximal	Rotational
Wrist	Hand down	Distal	Linear
Wrist	Hand up	Distal	Linear
Wrist	External rotation	Distal	Rotational
Wrist	Internal rotation	Distal	Rotational
Thumb	Open	Distal	Linear
Thumb	Close	Distal	Linear
Fingers	Open	Distal	Linear
Fingers	Close	Distal	Linear

**Table 3:** Summary presentation and description of the most important models and connectivity metrics or measures that were used in the methodological section of this study.

Metric or model name	Acronym	Description	
Current cortical density	CCD	Forward problem solution	A model that aims to explain the correspondence of cortical source activity to scalp electrical potentials, taking account of skull and scalp conductivity.
Weighted minimum norm estimate	wMNE	Inverse problem solution	An estimation of how signals captured at the scalp correspond to source activations, with their power limited by the cortical physiology.
Directed transfer function	DTF	Granger causality measure	A metric of effective network connectivity (functional connectivity that incorporates causal relations instead of statistical inference alone) that produces directed networks with weighted edges.
Characteristic path length	CPL	Network integration	A representative measure of shortest distances between network nodes that are connected to each other.
Clustering coefficient	CC	Network segregation	A measure of the tendency of network nodes to become organized into functionally separated clusters.
Density	*D*	Network density	A measure of existing connections against the theoretical maximum number of possible connections if the network was fully connected.
Small-worldness	SW	Overall network effectiveness	A model of network behavior, where short paths and increased forming of functional clusters lead to optimization and resilience of information transfer.
Out-strength and in-strength	OS and IS		The total nodal sum of weights from outgoing and incoming connections, respectively.

**Table 4:** Descriptive statistics of nodal incoming strengths with statistically significant differences between healthy and SCI participants and between-group comparison results.

Nodes	Median		Mean ranks		IQR: [Q_1_, Q_2_]		Healthy versus SCI
	Healthy	SCI	Healthy	SCI	Healthy	SCI	
SIF L	**0.235**	0.230	37.72	27.28	[0.231, 0.253]	[0.226, 0.238]	*U* = 345.0, *p* = 0.025
SIH L	**0.263**	0.246	44.13	20.88	[0.258, 0.270]	[0.239, 0.256]	*U* = 140.0, *p* < 0.001
SIH R	**0.256**	0.233	40.94	24.06	[0.243, 0.265]	[0.229, 0.248]	*U* = 242.0, *p* < 0.001
CMA L	0.410	**0.438**	18.84	46.16	[0.402, 0.419]	[0.431, 0.445]	*U* = 75.0, *p* < 0.001
CMA R	**0.486**	0.473	38.38	26.63	[0.475, 0.509]	[0.458, 0.489]	*U* = 324.0, *p* = 0.012
SMA L	**0.236**	0.217	41.34	23.66	[0.230, 0.243]	[0.213, 0.229]	*U* = 229.0, *p* < 0.001
SMA R	**0.305**	0.289	39.50	25.50	[0.296, 0.314]	[0.281, 0.297]	*U* = 288.0, *p* = 0.003
PMd L	**0.309**	0.272	46.84	18.16	[0.296, 0.320]	[0.260, 0.278]	*U* = 53.0, *p* < 0.001
PMd R	**0.299**	0.284	37.78	27.22	[0.290, 0.304]	[0.274, 0.299]	*U* = 343.0, *p* = 0.023
PMv L	**0.252**	0.232	40.53	24.47	[0.236, 0.259]	[0.220, 0.243]	*U* = 255.0, *p* = 0.001
PMv R	**0.262**	0.247	41.81	23.19	[0.254, 0.267]	[0.240, 0.255]	*U* = 214.0, *p* < 0.001

**Table 5:** Descriptive statistics of nodal outgoing strengths with statistically significant differences between healthy and SCI participants and between-group comparison results.

Nodes	Median		Mean ranks		IQR: [Q_1_, Q_2_]		Healthy versus SCI
	Healthy	SCI	Healthy	SCI	Healthy	SCI	
S1F L	0.073	**0.084**	21.34	43.66	[0.080, 0.095]	[0.080, 0.095]	*U* = 155.000, *p* < 0.001
S1F R	0.131	0.133	31.81	33.19	[0.123, 0.146]	[0.123, 0.146]	*U* = 490.000, *p* = 0.768
S1H L	**0.090**	0.084	38.38	26.63	[0.084, 0.100]	[0.077, 0.091]	*U* = 324.000, *p* = 0.012
S1H R	0.091	**0.166**	16.50	48.50	[0.086, 0.096]	[0.153, 0.181]	*U* = 0.000, *p* < 0.001
SAC L	0.071	**0.087**	18.28	46.72	[0.069, 0.074]	[0.084, 0.092]	*U* = 57.000, *p* < 0.001
SAC R	**0.025**	0.015	47.41	17.59	[0.023, 0.027]	[0.014, 0.016]	*U* = 35.000, *p* < 0.001
S2 L	0.010	**0.022**	17.41	47.59	[0.009, 0.012]	[0.020, 0.024]	*U* = 29.000, *p* < 0.001
S2 R	0.128	**0.148**	20.91	44.09	[0.124, 0.138]	[0.139, 0.159]	*U* = 141.000, *p* < 0.001
M1F L	**0.078**	0.073	38.13	26.88	[0.072, 0.088]	[0.069, 0.076]	*U* = 332.000, *p* = 0.016
M1F R	**0.294**	0.236	47.69	17.31	[0.286, 0.324]	[0.230, 0.263]	*U* = 26.000, *p* < 0.001
M1H L	**0.311**	0.170	47.59	17.41	[0.283, 0.339]	[0.163, 0.181]	*U* = 29.000, *p* < 0.001
M1H R	**0.061**	0.059	38.34	26.66	[0.059, 0.066]	[0.058, 0.061]	*U* = 325.000, *p* = 0.012
M1L L	**0.003**	0.003	26.25	38.75	[0.003, 0.003]	[0.003, 0.004]	*U* = 312.000, *p* = 0.007
M1L R	**0.011**	0.010	42.78	22.22	[0.010, 0.013]	[0.009, 0.010]	*U* = 183.000, *p* < 0.001
CMA L	**2.927**	2.519	47.28	17.72	[2.805, 3.008]	[2.404, 2.581]	*U* = 39.000, *p* < 0.001
CMA R	0.879	**1.156**	18.41	46.59	[0.784, 0.973]	[1.082, 1.261]	*U* = 61.000, *p* < 0.001
SMA L	**0.350**	0.276	46.25	18.75	[0.326, 0.375]	[0.267, 0.295]	*U* = 72.000, *p* < 0.001
SMA R	**0.206**	0.187	41.72	23.28	[0.191, 0.226]	[0.176, 0.194]	*U* = 217.000, *p* < 0.001
pSMA L	**0.044**	0.031	46.50	18.50	[0.040, 0.048]	[0.030, 0.035]	*U* = 64.000, *p* < 0.001
pSMA R	0.026	**0.031**	19.28	45.72	[0.024, 0.027]	[0.030, 0.033]	*U* = 89.000, *p* < 0.001
PMd L	0.149	**0.187**	21.13	43.88	[0.141, 0.167]	[0.179, 0.201]	*U* = 148.000, *p* < 0.001
PMd R	0.484	**0.577**	18.25	46.75	[0.448, 0.521]	[0.559, 0.606]	*U* = 56.000, *p* < 0.001
PMv L	0.014	**0.024**	16.50	48.50	[0.013, 0.014]	[0.022, 0.025]	*U* = 0.000, *p* < 0.001
PMv R	0.016	**0.032**	17.91	47.09	[0.014, 0.018]	[0.028, 0.035]	*U* = 45.000, *p* < 0.001

## Supplementary Materials

Supplementary Materials Statistical analysis: methodology and results for CPL, CC. D, SW, and correlations. Figure 1: SCI group shows increase in mean global clustering coefficient compared to healthy group in late time interval of the alpha networks, during “left,” “distal,” and “rotational” motor imagery categories. Figure 2: SCI group shows increased small-worldness (SW) compared to healthy group in the late time interval of alpha networks for “distal” imagery category. Figure 3: between-group differences in CMA_L were negatively correlated to those in CMA_R in early alpha walking and late beta walking. Table 1: descriptive statistics of total nodal out-strengths and in-strengths of the networks of the SCI subjects. Table 2: descriptive statistics of total nodal out-strengths and in-strengths of the networks of the healthy subjects.
