# Supplementary material for: Functional Brain Connectivity during Multiple Motor Imagery Tasks in Spinal Cord Injury
Source: Neural Plast. 2018 May 2;2018:9354207. doi: 10.1155/2018/9354207 (PMC5954936; doi:10.1155/2018/9354207)
Supplement: Supplementary Materials — Statistical analysis: methodology and results for CPL, CC. D, SW, and correlations. Figure 1: SCI group shows increase in mean global clustering coefficient compared to healthy group in late time interval of the alpha networks, during “left,” “distal,” and “rotational” motor imagery categories. Figure 2: SCI group shows increased small-worldness (SW) compared to healthy group in the late time interval of alpha networks for “distal” imagery category. Figure 3: between-group differences in CMA_L were negatively correlated to those in CMA_R in early alpha walking and late beta walking. Table 1: descriptive statistics of total nodal out-strengths and in-strengths of the networks of the SCI subjects. Table 2: descriptive statistics of total nodal out-strengths and in-strengths of the networks of the healthy subjects. [file 9354207.f1.pdf]

Functional Brain Connectivity during
Multiple Motor Imagery Tasks in Spinal Cord Injury

Supplementary material on statistical analysis of graph properties

Methodology

Initially, we planned within-group comparisons at each brain network characteristic (CC, CPL, D, S) using as grouping factor the time (early, late). Differences of variables at the two time conditions were computed and then explored for normality assumption. Normality assumption was investigated through different procedures such as formal normality test (Shapiro-Wilk test), visual inspection of histograms, boxplots and normal q-q plots as well as checking the skewness and kurtosis values. Depending on normality assumption, different analyses were planned for both groups (paired t-tests or Wilcoxon Signed Ranks tests). Subsequently, we ran within-group comparisons at each brain network characteristic grouping by the rhythm (alpha, beta). Differences of variables at the two rhythms were calculated and then explored for normality assumption using the aforementioned procedures.

With regards to between-group comparisons, we used the calculated differences of variables at the two time conditions and we performed either independent samples t-tests or Mann-Whitney U tests depending on the normality assumption.

Regarding IS and OS, those properties were calculated for every node of the network in alpha and beta networks and in early and late time intervals and averaged by imagery category. They were tested for normality assumption for both groups and analyzed within groups using descriptive statistics and between groups (SCI, Healthy) using Mann-Whitney U test. Strengths of nodes were visualized with bar-plots and targeted differences were tested, between nodes CMA_L and CMA_R, for statistical significance using either Pearson or Spearman correlation coefficient depending on normality assumption of targeted differences.

Results

- Mean clustering coefficient (CC)

In healthy participants, considerable decrease was found only in the mean CC of the left hand in the alpha band *(t=2.435, df=8, p=0.041; Early alpha left CC(mean): 0.0094; Late alpha left CC(mean): 0.0086).* While in SCI group, significant increase was observed in the mean CC of distal

movement in the alpha band *(t=-2.574, df=9, p=0.030; Early alpha distal CC(mean): 0.0076; Late alpha distal CC(mean): 0.0082).* Considerable differences in mean CC at the beta band were not found. When exploring within-group difference using as grouping factor the rhythm, both groups showed significant diminutions in mean CC at beta rhythm compared to alpha rhythms across almost all tasks and in both time conditions (early, late). More precisely, mean CC in SCI subjects was significantly decreased at beta rhythm compared to alpha across all tasks in late movements *(Late alpha hands – Late beta hands: t=6.349, df=9, p<0.001; Late alpha hands(mean):0.0082, Late beta hands(mean): 0.0065; Late alpha left – Late beta left: t=4.879, df=9, p=0.001; Late alpha left(mean):0.0080, Late beta left(mean): 0.0064; Late alpha right – Late beta right: t=7.293, df=9, p<0.001; Late alpha right(mean):0.0084, Late beta right(mean): 0.0066; Late alpha distal – Late beta distal: t=7.439, df=9, p<0.001; Late alpha distal (mean):0.0082, Late beta distal(mean): 0.0063; Late alpha proximal – Late beta proximal: t=3.941, df=9, p=0.003; Late alpha proximal(mean):0.0082, Late beta proximal(mean): 0.0067; Late alpha linear – Late beta linear: t=6.150, df=9, p<0.001; Late alpha linear(mean):0.0081, Late beta linear(mean): 0.0064; Late alpha rotational – Late beta rotational: t=3.829, df=9, p=0.004; Late alpha rotational(mean):0.0085, Late beta rotational(mean): 0.0068; Late alpha walking – Late beta walking: t=2.316, df=9, p=0.046; Late alpha walking (mean):0.0088, Late beta walking (mean): 0.0071).* Similarly to late movements, SCI group showed considerable decreased in early movements across all task apart from the walking task *(Early alpha hands – Early beta hands: t=5.022, df=9, p=0.001; Early alpha hands(mean):0.0078, Early beta hands(mean): 0.0065; Early alpha left – Early beta left: t=4.078, df=9, p=0.003; Early alpha left(mean):0.0078, Early beta left(mean): 0.0065; Early alpha right– Early beta right: t=5.052, df=9, p=0.001; Early alpha right (mean):0.0078, Early beta right(mean): 0.0065; Early alpha distal– Early beta distal: t=5.010, df=9, p=0.001; Early alpha distal (mean):0.0076, Early beta distal(mean): 0.0064; Early alpha proximal– Early beta proximal: t=4.264, df=9, p=0.002; Early alpha proximal (mean):0.0080, Early beta proximal(mean): 0.0066; Early alpha linear– Early beta linear: t=4.533, df=9, p=0.001; Early alpha linear (mean):0.0078, Early beta linear(mean): 0.0065; Early alpha rotational–Early beta rotational: t=5.141, df=9, p=0.001; Early alpha rotational (mean):0.0079, Early beta rotational (mean): 0.0067; Early alpha walking–Early beta walking: t=0.279, df=9, p=0.790; Early alpha walking (mean):0.0063, Early beta walking (mean): 0.0061).*

In healthy participants we found statistically significant decreases in mean CC at beta across all tasks in the first time condition (early) *(Early alpha hands – Early beta hands: t=5.479, df=8, p=0.001; Early alpha hands(mean):0.0090, Early beta hands(mean): 0.0066; Early alpha left – Early beta left: t=5.114, df=8, p=0.001; Early alpha left(mean):0.0095, Early beta left(mean): 0.0068; Early alpha right– Early beta right: z=-2.666, p=0.008; Early alpha right (median):0.0074, Early beta right(median): 0.0058; Early alpha distal– Early beta distal: z=-2.666, p=0.008; Early alpha distal (median):0.0080, Early beta distal(median): 0.0057; Early alpha proximal– Early beta proximal: t=6.442, df=8, p<0.001; Early alpha proximal (mean):0.0083, Early beta proximal(mean): 0.0062; Early alpha linear–Early beta linear: z=-2.666, p=0.008; Early alpha linear (median):0.0074, Early beta linear(median): 0.0056; Early alpha rotational–Early beta rotational: t=5.205, df=8, p=0.001; Early alpha rotational (mean):0.0095, Early beta rotational (mean): 0.0066; Early alpha walking–Early beta walking: t=2.524, df=8, p=0.036; Early alpha walking (mean):0.0084, Early beta walking (mean): 0.0064).* We found similar outcomes in late condition of all task apart from the walking task which did not reach statistical significance *(Late alpha hands – Late beta hands: t=4.965, df=8, p=0.001; Late alpha hands(mean):0.0089, Late beta hands(mean): 0.0067; Late alpha left – Late beta left: t=4.739, df=8, p=0.001; Late alpha left(mean):0.0086, Late beta left(mean): 0.0066; Late alpha right – Late beta right: t=4.468, df=8, p=0.002; Late alpha right(mean):0.0092, Late beta right(mean): 0.0069; Late*

alpha distal – Late beta distal: t=4.324, df=8, p=0.003; Late alpha distal (mean):0.0087, Late beta distal(mean): 0.0068; Late alpha proximal – Late beta proximal: t=4.862, df=8, p=0.001; Late alpha proximal(mean):0.0091, Late beta proximal(mean): 0.0066; Late alpha linear – Late beta linear: t=4.523, df=8, p=0.002; Late alpha linear(mean):0.0091, Late beta linear(mean): 0.0068; Late alpha rotational – Late beta rotational: t=5.359, df=8, p=0.001; Late alpha rotational(mean):0.0084, Late beta rotational(mean): 0.0065; Late alpha walking – Late beta walking: z=-1.125, p=0.260; Late alpha walking (median):0.0075, Late beta walking (median): 0.0062).

Between-group comparisons revealed significant changes in the mean CC in the left movements *(t=2.672, df=17, p=0.016),* in rotational movements *(t=2.104, df=17, p=0.051*) and distal movements *(U=20.00, p=0.041),* all in alpha band. In more detail, SCI subjects seem to show increase in mean CC of alpha network in the late part of the aforementioned movements *(Alpha left dif (mean) – SCI: 0.00019, Healthy: -0.00084; Alpha distal dif (median) – SCI: 0.00049, Healthy: -0.00075; Alpha rotational dif (mean) – SCI: 0.00061, Healthy: -0.0010)* (Figure 1). Significant differences were not found between groups in the beta networks of all tasks.

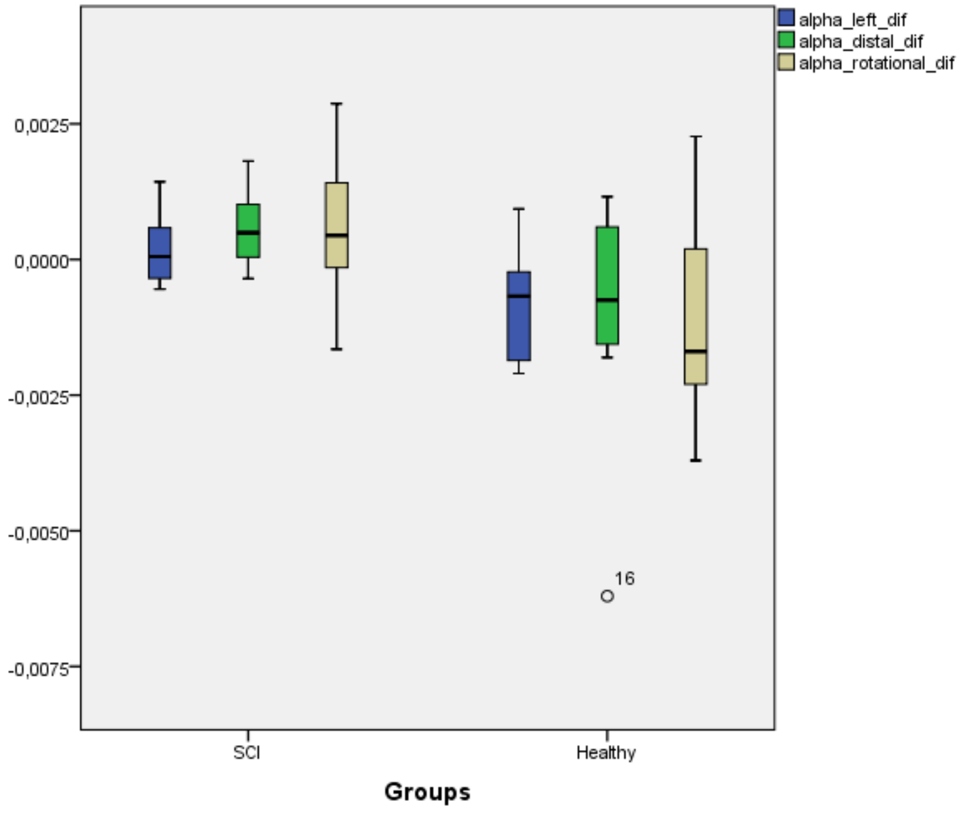

Figure 1: SCI group shows increase in mean global clustering coefficient compared to healthy group in late time interval of the alpha networks, during «left», «distal» and «rotational» motor imagery categories.

- Mean Characteristic Path Length (CPL)

Significant difference was shown only in mean CPL of SCI group's alpha band at the walking task *(t=2.743, df=9, p=0.023).* More precisely, SCI participants were characterized by a decrease in mean CPL at the second stage of the task (late) *(Early alpha walking CPL(mean): 7.809; Late alpha*

left CPL(mean): 7.032). Changes in the CPL in the beta rhythm comparing the two time stages were not observed. Healthy subjects did not significant alter their mean CPL at the two time stages of different movement tasks.

Comparing the mean CPL between rhythms (alpha, beta) in both groups, significant decreases were observed in SCI group in both early and late movement of almost all tasks. Differences between rhythms in the late movement of walking task did not reach significance *(Early alpha hands – Early beta hands: t=5.680, df=9, p<0.001; Early alpha hands(mean):7.3680, Early beta hands(mean): 6.5512; Early alpha left – Early beta left: t=5.274, df=9, p=0.001; Early alpha left(mean):7.3596, Early beta left(mean): 4.4919; Early alpha right– Early beta right: t=5.842, df=9, p<0.001; Early alpha right (mean):7.3764, Early beta right(mean): 6.6104; Early alpha distal–Early beta distal: t=6.454, df=9, p<0.001; Early alpha distal (mean):7.3921, Early beta distal(mean): 6.5670; Early alpha proximal–Early beta proximal: t=4.789, df=9, p=0.001; Early alpha proximal (mean):7.3439, Early beta proximal(mean): 6.5353; Early alpha linear–Early beta linear: t=5.425, df=9, p<0.001; Early alpha linear (mean):7.3333, Early beta linear(mean): 6.5418; Early alpha rotational–Early beta rotational: z=-2.803 p=0.005; Early alpha rotational (median):7.2648, Early beta rotational (median): 6.6480; Early alpha walking–Early beta walking: z=-2.803, p=0.005; Early alpha walking (median):7.8560, Early beta walking (median): 6.7650; Late alpha hands – Late beta hands: t=6.994, df=9, p<0.001; Late alpha hands(mean):7.2398, Late beta hands(mean): 6.5754; Late alpha left – Late beta left: t=6.740, df=9, p<0.001; Late alpha left(mean):7.3402, Late beta left(mean): 6.5718; Late alpha right – Late beta right: t=4.688, df=9, p=0.001; Late alpha right(mean):7.1394, Late beta right(mean): 6.5790; Late alpha distal – Late beta distal: t=5.593, df=9, p<0.001; Late alpha distal (mean):7.2137, Late beta distal(mean): 6.5899; Late alpha proximal – Late beta proximal: z=-2.503, p=0.005; Late alpha proximal(median):7.2701, Late beta proximal(median): 6.5438; Late alpha linear – Late beta linear: t=5.444, df=9, p<0.001; Late alpha linear(mean):7.2496, Late beta linear(mean): 6.5936; Late alpha rotational – Late beta rotational: t=5.698, df=9, p<0.001; Late alpha rotational(mean):7.2102, Late beta rotational(mean): 6.5208; Late alpha walking – Late beta walking: t=2.014, df=9, p=0.075; Late alpha walking (mean):7.0320, Late beta walking (mean): 6.4858).*

Healthy group showed considerable diminution in mean CPL in beta rhythm compared to alpha rhythm across all task and in both time conditions (early, late) *(Early alpha hands – Early beta hands: t=4.999, df=8, p=0.001; Early alpha hands(mean):7.2371, Early beta hands(mean): 6.6192; Early alpha left – Early beta left: t=4.430, df=8, p=0.002; Early alpha left(mean):7.2209, Early beta left(mean): 6.5732; Early alpha right– Early beta right: t=4.924, df=8, p=0.001; Early alpha right (mean):7.2532, Early beta right(mean): 6.6652; Early alpha distal– Early beta distal: t=3.129, df=8, p=0.014; Early alpha distal (mean):7.2024, Early beta distal(mean): 6.6165; Early alpha proximal– Early beta proximal: t=5.852, df=8, p<0.001; Early alpha proximal (mean):7.2717, Early beta proximal(mean): 6.6219; Early alpha linear–Early beta linear: t=5.530, df=8, p=0.001; Early alpha linear (mean):7.2889, Early beta linear(mean): 6.6352; Early alpha rotational–Early beta rotational: t=2.273, df=8, p=0.053 (marginal); Early alpha rotational (mean):7.0815, Early beta rotational (mean): 6.5711; Early alpha walking–Early beta walking: t=3.241, df=8, p=0.012; Early alpha walking (mean):7.3981, Early beta walking (mean): 6.3506;*

Late alpha hands – Late beta hands: t=5.216, df=8, p=0.001; Late alpha hands(mean):7.2593, Late beta hands(mean): 6.5992; Late alpha left – Late beta left: t=6.844, df=8, p<0.001; Late alpha left(mean):7.3091, Late beta left(mean): 6.5792; Late alpha right – Late beta right: t=3.645, df=8, p=0.007; Late alpha right(mean):7.2095, Late beta right(mean): 6.6192; Late alpha distal – Late beta distal: t=7.349, df=8, p<0.001; Late alpha distal (mean):7.3312, Late beta distal(mean): 6.5633; Late alpha proximal – Late beta proximal: t=3.408, df=8, p=0.009; Late alpha proximal(mean):7.1874, Late beta proximal(mean): 6.6352; Late alpha linear

– Late beta linear: t=5.246, df=8, p=0.001; Late alpha linear(mean):7.2205, Late beta linear(mean): 6.6018; Late alpha rotational – Late beta rotational: t=4.478, df=8, p=0.002; Late alpha rotational(mean):7.3756, Late beta rotational(mean): 6.5914; Late alpha walking – Late beta walking: t=4.303, df=8, p=0.003; Late alpha walking (median):7.8567, Late beta walking (median): 6.6091).

Planned between-group comparisons did not reveal any considerable difference in both networks across all tasks *(Alpha: Hands: t=-1.350, df=17, p=0.195; Left: t=-0.768, df=17, p=0.453; Right: t=-1.062, df=17, p=0.303; Distal: t=-1.706, df=17, p=0.106; Proximal: t=0.044, df=17, p=0.966; Linear: t=-0.120, df=17, p=0.906; Rotational: U=23.00, p=0.072; Walking: t=-1.869, df=11.512, p=0.087; Beta - Hands: t=1.133, df=17, p=0.273; Left: t=1.091, df=17, p=0.291; Right: t=0.233, df=17, p=0.819; Distal: t=1.315, df=17, p=0.206; Proximal: t=0.219, df=17, p=0.829; Linear: t=1.732, df=17, p=0.101; Rotational: t=-1.097, df=17, p=0.288; Walking: t=-1.799, df=17, p=0.090).*

- Density (DE)

Healthy group showed a significant increase of density at the late stage of linear movements in alpha rhythm *(t=-2.543, df=8, p=0.035; Early alpha linear DE(mean): 0.3595; Late alpha linear DE(mean): 0.3713)* whereas density was considerably decreased at the late stage of proximal movements in beta rhythm *(t=3.038, df=8, p=0.016; Early beta proximal DE(mean): 0.5904; Late beta proximal DE(mean): 0.5784)*. SCI subjects increased their density when comparing the two time conditions of different tasks of the right hand in alpha band *(t=-2.962, df=9, p=0.016; Early alpha right DE(mean): 0.3663; Late alpha right DE(mean): 0.3801)*. Alterations in density the beta rhythm across different tasks were not found.

The density of both groups was significantly increased in beta rhythm relative to alpha at both early and late movement of all tasks *(SCI: Early alpha hands – Early beta hands: t=-16.826, df=9, p<0.001; Early alpha hands(mean):0.3757, Early beta hands(mean): 0.5975; Early alpha left – Early beta left: t=-14.970, df=9, p<0.001; Early alpha left(mean):0.3850, Early beta left(mean):0.6094; Early alpha right– Early beta right: t=-18.596, df=9, p<0.001; Early alpha right (mean):0.3663, Early beta right(mean):0.5856; Early alpha distal–Early beta distal: t=-17.191, df=9, p<0.001; Early alpha distal (mean):0.3778, Early beta distal(mean):0.5975; Early alpha proximal–Early beta proximal: t=-15.879, df=9, p<0.001; Early alpha proximal (mean):0.3735, Early beta proximal(mean):0.5975; Early alpha linear–Early beta linear: t=-16.619, df=9, p<0.001; Early alpha linear (mean):0.3806, Early beta linear(mean):0.6015; Early alpha rotational–Early beta rotational: t=-15.094, df=9, p<0.001; Early alpha rotational (mean):0.3608, Early beta rotational (mean): 0.5857; Early alpha walking–Early beta walking: t=-12.999, df=9, p<0.001; Early alpha walking (mean):0.3697, Early beta walking (mean): 0.5942; Late alpha hands – Late beta hands: t=-19.839, df=9, p<0.001; Late alpha hands(mean):0.3824, Late beta hands(mean): 0.5982; Late alpha left – Late beta left: t=-21.138, df=9, p<0.001; Late alpha left(mean): 0.3847, Late beta left(mean): 0.6039; Late alpha right – Late beta right: t=-16.241, df=9, p<0.001; Late alpha right(mean): 0.3801, Late beta right(mean):0.5925; Late alpha distal – Late beta distal: t=-19.181, df=9, p<0.001; Late alpha distal (mean):0.3840, Late beta distal(mean): 0.6015; Late alpha proximal – Late beta proximal: t=-17.945,df=9, p<0.001; Late alpha proximal(mean):0.3808, Late beta proximal(mean): 0.5948; Late alpha linear – Late beta linear: t=-18.035, df=9, p<0.001; Late alpha linear(mean):0.3834, Late beta linear(mean): 0.6004; Late alpha rotational – Late beta rotational: t=-16.972, df=9, p<0.001; Late alpha*

rotational(mean):0.5913, Late beta rotational(mean): 6.5208; Late alpha walking – Late beta walking: t=-7.383, df=9, p<0.001; Late alpha walking (mean):0.3812, Late beta walking (mean): 0.5964;

Healthy: Early alpha hands – Early beta hands: t=-33.561, df=8, p<0.001; Early alpha hands(mean):0.3600, Early beta hands(mean): 0.5806; Early alpha left – Early beta left: t=-23.569, df=8, p<0.001; Early alpha left(mean):0.3601, Early beta left(mean):0.5830; Early alpha right– Early beta right: t=-37.196, df=8, p<0.001; Early alpha right (mean):0.3599, Early beta right(mean):0.5781; Early alpha distal–Early beta distal: t=-23.734, df=8, p<0.001; Early alpha distal (mean):0.3557, Early beta distal(mean):0.5707; Early alpha proximal–Early beta proximal: t=-34.544, df=8, p<0.001; Early alpha proximal (mean):0.3643, Early beta proximal(mean):0.5904; Early alpha linear–Early beta linear: t=-31.715, df=8, p<0.001; Early alpha linear (mean):0.3595, Early beta linear(mean):0.5772; Early alpha rotational–Early beta rotational: t=-17.871, df=8, p<0.001; Early alpha rotational (mean):0.3615, Early beta rotational (mean): 0.5905; Early alpha walking–Early beta walking: t=-10.401, df=8, p<0.001; Early alpha walking (mean):0.3500, Early beta walking (mean): 0.5918; Late alpha hands – Late beta hands: t=-41.143, df=8, p<0.001; Late alpha hands(mean):0.3679, Late beta hands(mean): 0.5817; Late alpha left – Late beta left: t=-45.504, df=8, p<0.001; Late alpha left(mean): 0.3649, Late beta left(mean): 0.5891; Late alpha right – Late beta right: t=-31.302, df=8, p<0.001; Late alpha right(mean): 0.3710, Late beta right(mean):0.5743; Late alpha distal – Late beta distal: t=-41.975, df=8, p<0.001; Late alpha distal (mean):0.3676, Late beta distal(mean): 0.5850; Late alpha proximal – Late beta proximal: t=-31.840, df=8, p<0.001; Late alpha proximal(mean):0.3683, Late beta proximal(mean): 0.5784; Late alpha linear – Late beta linear: t=-41.478, df=8, p<0.001; Late alpha linear(mean):0.3713, Late beta linear(mean): 0.5815; Late alpha rotational – Late beta rotational: t=-26.578, df=9, p<0.001; Late alpha rotational(mean):0.3577, Late beta rotational(mean): 0.5821; Late alpha walking – Late beta walking: t=-8.940, df=8, p<0.001; Late alpha walking (mean):0.2915, Late beta walking (mean): 0.5644).

Planned between-group comparisons did not reveal any considerable difference in both networks across all tasks *(Alpha: Hands: t=-0.189, df=17, p=0.852; Left: t=-0.445, df=17, p=0.662; Right: t=0.367, df=17, p=0.718; Distal: t=-0.554, df=17, p=0.587; Proximal: t=0.314, df=17, p=0.758; Linear: t=-1.151, df=17, p=0.266; Rotational: t=1.366, df=17, p=0.190; Walking: t=1.411, df=17, p=0.176; Beta - Hands: t=-0.072, df=17, p=0.943; Left: U=26.50 p=0.131; Right: t=1.244, df=17, p=0.230; Distal: t=-1.042, df=17, p=0.312; Proximal: t=1.357, df=17, p=0.193; Linear: t=-0.696, df=17, p=0.496; Rotational: t=1.504, df=17, p=0.151; Walking: t=0.600, df=17, p=0.557).*

- Small-world-ness (SW)

Significant results have found in small-world metric of healthy participants at distal movements in both alpha and beta rhythms *(alpha: t=2.201, df=8, p=0.059; beta: t=3.044, df=8, p=0.016).* In more detail, considerable decrease in small-world-ness was observed in distal movements on both brain rhythms *(Early alpha distal SW(mean): 1.553; Late alpha distal SW(mean): 1.406; Early beta distal SW(mean): 1.159; Late beta distal SW(mean): 1.137).*While in SCI group considerable differences in SW across different tasks and in both rhythms were not observed.

SCI participants showed decreased small-world-ness in beta relative to alpha at the late movement of all tasks *(Late alpha hands – Late beta hands: t=5.589, df=9, p<0.001; Late alpha hands(mean):1.3809, Late beta hands(mean): 1.1190; Late alpha left – Late beta left: t=5.898, df=9, p<0.001;*

Late alpha left(mean): 1.3596, Late beta left(mean): 1.1094; Late alpha right – Late beta right: z=-2.803, p=0.005; Late alpha right(median): 1.3077, Late beta right(median):1.0744; Late alpha distal – Late beta distal: t=6.209, df=9, p<0.001; Late alpha distal (mean):1.3811, Late beta distal(mean): 1.1108; Late alpha proximal – Late beta proximal: t=4.815, df=9, p=0.001; Late alpha proximal(mean):1.3806, Late beta proximal(mean): 1.1271; Late alpha linear – Late beta linear: t=6.049, df=9, p<0.001; Late alpha linear(mean):1.3669, Late beta linear(mean): 1.1067; Late alpha rotational – Late beta rotational: z=-2.803, p=0.005; Late alpha rotational(median):1.2848, Late beta rotational(median): 1.0961; Late alpha walking – Late beta walking: t=3.341, df=9, p=0.009; Late alpha walking (mean):1.4859, Late beta walking (mean): 1.1691). Similarly, we observed significantly lower SW values at the early movement of almost all tasks apart from the walking *Early alpha hands – Early beta hands: t=5.498, df=9, p<0.001; Early alpha hands(mean):1.3815, Early beta hands(mean): 1.1240; Early alpha left – Early beta left: t=4.831, df=9, p=0.001; Early alpha left(mean):1.3310, Early beta left(mean):1.1124; Early alpha right– Early beta right: t=5.764, df=9, p<0.001; Early alpha right (mean):1.4320, Early beta right(mean):1.1356; Early alpha distal–Early beta distal: z=-2.803, p=0.005; Early alpha distal (median):1.2717, Early beta distal(median):1.0838; Early alpha proximal–Early beta proximal: t=4.993, df=9, p=0.001; Early alpha proximal (mean):1.4072, Early beta proximal(mean):1.1275; Early alpha linear–Early beta linear: t=5.068, df=9, p=0.001; Early alpha linear (mean):1.3787, Early beta linear(mean):1.1137; Early alpha rotational–Early beta rotational: t=6.432, df=9, p<0.001; Early alpha rotational (mean):1.3900, Early beta rotational (mean): 1.1549; Early alpha walking–Early beta walking: t=1.835, df=9, p=0.100; Early alpha walking (mean):1.2259, Early beta walking (mean): 1.0933)*.

In line with previous findings, mean SW was also considerably decreased in beta band of healthy participants compared to alpha at the two time conditions of all tasks *Early alpha hands – Early beta hands: t=4.342, df=8, p=0.002; Early alpha hands(mean):1.4900, Early beta hands(mean): 1.1276; Early alpha left – Early beta left: t=4.518, df=8, p=0.002; Early alpha left(mean):1.5100, Early beta left(mean):1.1315; Early alpha right– Early beta right: z=-2.666, p=0.008; Early alpha right (median):1.2906, Early beta right(median):1.0924; Early alpha distal–Early beta distal: t=3.950, df=8, p=0.004; Early alpha distal (mean):1.5530, Early beta distal(mean):1.1587; Early alpha proximal–Early beta proximal: z=-2.666, p=0.008; Early alpha proximal (median):1.2785, Early beta proximal(median):1.0804; Early alpha linear–Early beta linear: t=4.886, df=8, p=0.001; Early alpha linear (mean):1.4645, Early beta linear(mean):1.1301; Early alpha rotational–Early beta rotational: z=-2.666, p=0.008; Early alpha rotational (median):1.3649, Early beta rotational (median): 1.0916; Early alpha walking–Early beta walking: t=2.335, df=8, p=0.048; Early alpha walking (mean):1.4189, Early beta walking (mean): 1.1157; Late alpha hands – Late beta hands: t=4.943, df=8, p=0.001; Late alpha hands(mean):1.4352, Late beta hands(mean): 1.1285; Late alpha left – Late beta left: t=4.066, df=8, p=0.004; Late alpha left(mean): 1.4514, Late beta left(mean): 1.1139; Late alpha right – Late beta right: t=5.618, df=8, p<0.001; Late alpha right(mean): 1.4190, Late beta right(mean):1.1431; Late alpha distal – Late beta distal: t=5.397, df=8, p=0.001; Late alpha distal (mean):1.4064, Late beta distal(mean): 1.1365; Late alpha proximal – Late beta proximal: t=4.219, df=8, p=0.003; Late alpha proximal(mean):1.4640, Late beta proximal(mean): 1.1204; Late alpha linear – Late beta linear: t=4.938, df=8, p=0.001; Late alpha linear(mean):1.4342, Late beta linear(mean): 1.1272; Late alpha rotational – Late beta rotational: z=-2.666, p=0.008; Late alpha rotational(median):1.3354, Late beta rotational(median): 1.0748; Late alpha walking – Late beta walking: z=-2.192, p=0.028; Late alpha walking (median):1.4593, Late beta walking (median): 1.1176).*

SCI group seem to have only a significant change in mean SW of alpha network while performing VMI on distal movements compared to healthy (*t=2.365, df=17, p=0.030).* More precisely, SCI group seems to show increased SW of alpha networks in the late part of the

distal movements *(Alpha distal dif (mean) – SCI: 0.0253, Healthy: -0.1467) (Figure 2).* Other between-group differences in the mean SW of both networks were not observed *(Alpha: Hands: U=39.00 p=0.624; Left: t=1.869, df=17, p=0.079; Right: U =43.00, p=0.870; Proximal: U =43.00, p=0.870; Linear: t=0.706, df=17, p=0.490; Rotational: t=1.404, df=17, p=0.178; Walking: U =39.00, p=0.624; Beta - Hands: U =43.00, p=0.870; Left: t=1.329, df=17, p=0.202; Right: U=36.00, p=0.462; Distal: t=1.294, df=17, p=0.213; Proximal: U=26.00, p=0.121; Linear: t=-0.362, df=17, p=0.722; Rotational: U=44.00, p=0.935; Walking: t=0.736, df=17, p=0.472).*

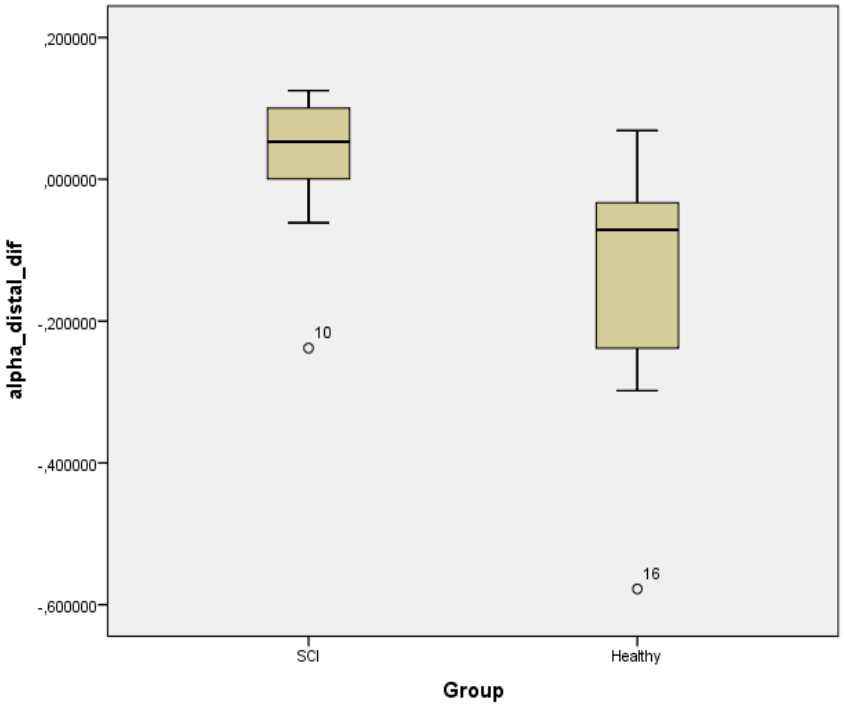

Figure 2: SCI group shows increased small-worldness (SW) compared to healthy group in the late time interval of alpha networks for «distal» imagery category.

- Correlations (OS, IS)

Between-group differences in targeted movements were computed in both CMA_L and CMA_R nodes. Possible significant correlations of between-group differences at the two nodes (CMA_L, CMA_R) were explored using either Pearson or Spearman depending on normality assumption of differences at each node.

Between-group differences in CMA_L were negatively correlated to those in CMA_R in targeted movements (early alpha walking *(r=-0.867, p=0.002)*, late alpha walking *(r=-0.250 p=0.517)*, early beta walking *(r=-0.502, p=0.169)*, late beta walking*(r=-0.827, p=0.006)*). However, statistical significance was reached only at early alpha walking and late beta walking tasks.

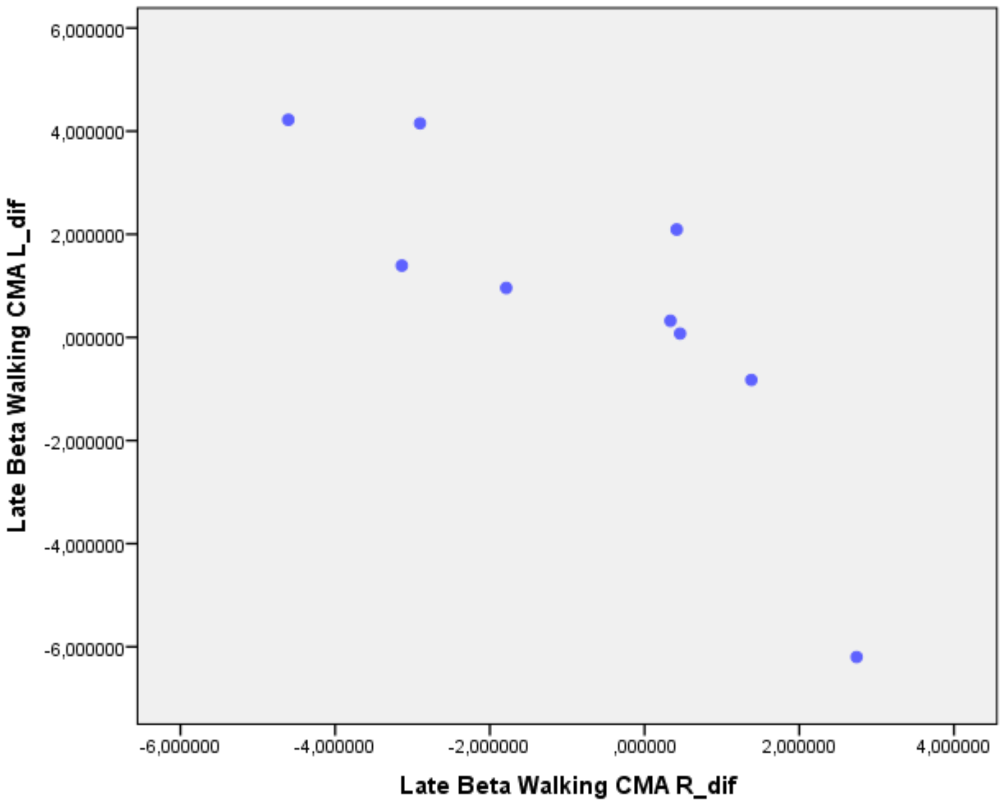

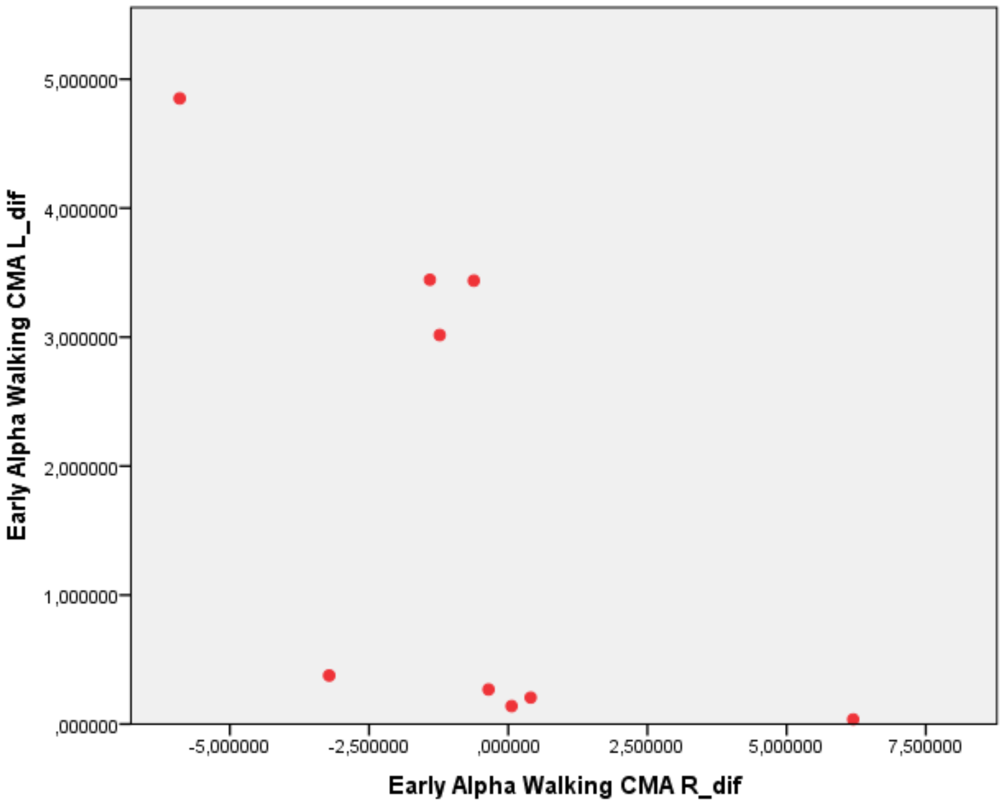

Figure 3: Between-group differences in CMA_L were negatively correlated to those in CMA_R in early alpha walking ($r=-0.867$, $p=0.002$) and late beta walking($r=-0.827$, $p=0.006$)).

Out-Strength SCI group			
**Nodes**	Median	Interquartile range (IQR)	
		25	75
**S1F_L**	0,083595	0,079982	0,094869
**S1F_R**	0,133132	0,122559	0,146268
**S1H_L**	0,084462	0,077372	0,0907
**S1H_R**	0,165826	0,153399	0,18122
**SAC_L**	0,086632	0,083832	0,092266
**SAC_R**	0,015154	0,014095	0,016102
**S2_L**	0,021811	0,019823	0,023619
**S2_R**	0,147513	0,138791	0,159253
**M1F_L**	0,072804	0,068563	0,076437
**M1F_R**	0,235897	0,229755	0,263421
**M1H_L**	0,170192	0,163242	0,180545
**M1H_R**	0,058814	0,057646	0,061025
**M1L_L**	0,003477	0,003265	0,003556
**M1L_R**	0,009681	0,009161	0,010249
**CMA_L**	2,518835	2,404343	2,580845
**SMA_L**	0,276298	0,267056	0,294762
**pSMA_L**	0,030927	0,030359	0,034996
**CMA_R**	1,156272	1,081914	1,261116
**SMA_R**	0,186739	0,176221	0,194128
**pSMA_R**	0,031388	0,030253	0,03321
**PMd_L**	0,187142	0,178956	0,200744
**PMd_R**	0,576984	0,559172	0,606098
**PMv_L**	0,023692	0,021851	0,025407
**PMv_R**	0,031872	0,028408	0,034923

In-Strength SCI group			
**Nodes**	Median	Interquartile Range	
		25	75
**S1F_L**	0.229646	0.226077	0.238457
**S1F_R**	0.266537	0.254689	0.27506
**S1H_L**	0.245518	0.239067	0.255876
**S1H_R**	0.233442	0.229398	0.247817
**SAC_L**	0.290414	0.278427	0.299449
**SAC_R**	0.302619	0.29323	0.318702
**S2_L**	0.230194	0.216279	0.241523
**S2_R**	0.252319	0.246569	0.265061
**M1F_L**	0.225068	0.219234	0.241234
**M1F_R**	0.233534	0.227704	0.242634
**M1H_L**	0.220557	0.215177	0.228598
**M1H_R**	0.231631	0.221952	0.239266
**M1L_L**	0.226648	0.209842	0.235824
**M1L_R**	0.228881	0.220792	0.239103
**CMA_L**	0.437572	0.430968	0.444785
**SMA_L**	0.21675	0.212613	0.229196
**pSMA_L**	0.232446	0.218799	0.247928
**CMA_R**	0.472534	0.458167	0.488587

**SMA_R**	0.289453	0.280492	0.296902
**pSMA_R**	0.234079	0.225829	0.244447
**PMd_L**	0.271741	0.260086	0.277511
**PMd_R**	0.284278	0.274103	0.298797
**PMv_L**	0.23209	0.219816	0.242863
**PMv_R**	0.247037	0.240143	0.254945

Table 1: Descriptive statistics of total nodal out-strengths and ins-strengths of the networks of the SCI subjects.

Out-Strength Healthy group			
**Nodes**	Median	Interquartile Range (IQR)	
		25	75
**S1F_L**	0,07276664	0,068599833	0,077988254
**S1F_R**	0,131157908	0,123140731	0,144792297
**S1H_L**	0,089526193	0,083922519	0,100419714
**S1H_R**	0,091449259	0,085894535	0,095590192
**SAC_L**	0,071107868	0,068730009	0,073922321
**SAC_R**	0,024705755	0,02250168	0,027266182
**S2_L**	0,009941762	0,009345575	0,012062296
**S2_R**	0,128416834	0,124224513	0,138433189
**M1F_L**	0,077637918	0,072164828	0,088331982
**M1F_R**	0,294097639	0,285836195	0,323900209
**M1H_L**	0,31130385	0,283424843	0,338668919
**M1H_R**	0,060623757	0,05924162	0,065829478
**M1L_L**	0,003069891	0,002877913	0,003443866
**M1L_R**	0,011300406	0,010473714	0,012648678
**CMA_L**	2,926688178	2,804858739	3,007724356
**SMA_L**	0,350342669	0,325683888	0,375285886
**pSMA_L**	0,043557658	0,040100233	0,048342254
**CMA_R**	0,878864391	0,784188978	0,972625683
**SMA_R**	0,20629489	0,191275664	0,225833894
**pSMA_R**	0,026230486	0,023711042	0,027071889
**PMd_L**	0,149297078	0,140656602	0,167157244
**PMd_R**	0,483959376	0,448350107	0,520612958
**PMv_L**	0,013772115	0,012639098	0,014493194
**PMv_R**	0,015689744	0,013646365	0,017669169

In-Strength Healthy group			
**Nodes**	Median	Interquartile Range (IQR)	
		25	75
**S1F_L**	0.235349	0.230972787	0.252504207
**S1F_R**	0.266194	0.261746828	0.275012169
**S1H_L**	0.263416	0.258416615	0.270243997
**S1H_R**	0.256461	0.242564442	0.264824323
**SAC_L**	0.290354	0.281284843	0.306082733
**SAC_R**	0.301615	0.290096972	0.309216962
**S2_L**	0.240973	0.226284016	0.249762059

**S2_R**	0.249813	0.245616657	0.257433804
**M1F_L**	0.229812	0.214459766	0.237064334
**M1F_R**	0.228883	0.213364643	0.239557715
**M1H_L**	0.227284	0.217648368	0.251065557
**M1H_R**	0.223288	0.213319375	0.234882479
**M1L_L**	0.230397	0.215067241	0.239285808
**M1L_R**	0.225567	0.220347875	0.234957581
**CMA_L**	0.40974	0.401661328	0.419015833
**SMA_L**	0.23645	0.229551037	0.243376293
**pSMA_L**	0.227776	0.219282951	0.240115818
**CMA_R**	0.485967	0.474933796	0.508912869
**SMA_R**	0.305023	0.296161205	0.314191822
**pSMA_R**	0.230512	0.221195221	0.246913071
**PMd_L**	0.308836	0.29592202	0.320410182
**PMd_R**	0.298981	0.2901009	0.304385703
**PMv_L**	0.251853	0.23634607	0.259327558
**PMv_R**	0.262244	0.253902082	0.266996539

Table 2: Descriptive statistics of total nodal out-strengths and ins-strengths of the networks of the Healthy subjects.
